# A macrophage-collagen fragment axis mediates subcutaneous adipose tissue remodeling in mice

**DOI:** 10.1073/pnas.2313185121

**Published:** 2024-02-01

**Authors:** Milica Vujičić, Isabella Broderick, Pegah Salmantabar, Charlène Perian, Jonas Nilsson, Carina Sihlbom Wallem, Ingrid Wernstedt Asterholm

**Affiliations:** ^a^Department of Physiology/Metabolic Physiology, Institute of Neuroscience and Physiology, The Sahlgrenska Academy at University of Gothenburg, Gothenburg 405 30, Sweden; ^b^Proteomics Core Facility, The Sahlgrenska Academy at University of Gothenburg, Gothenburg 405 30, Sweden

**Keywords:** subcutaneous adipose tissue, macrophages, CD206, collagen, fibrosis

## Abstract

Efficient removal of fibrillar collagen is essential for adaptive subcutaneous adipose tissue (SAT) expansion that protects against ectopic lipid deposition during weight gain. Here, we used mice to further define the mechanism for this collagenolytic process. We show that loss of collagen type-1 (CT1) and increased CT1-fragment levels in expanding SAT are associated with proliferation of resident M2-like macrophages that display increased CD206-mediated engagement in collagen endocytosis compared to chow-fed controls. Blockage of CD206 during acute high-fat diet-induced weight gain leads to SAT CT1-fragment accumulation associated with elevated inflammation and fibrosis markers. Moreover, these SAT macrophages’ engagement in collagen endocytosis is diminished in obesity associated with elevated levels collagen fragments that are too short to assemble into triple helices. We show that such short fragments provoke M2-macrophage proliferation and fibroinflammatory changes in fibroblasts. In conclusion, our data delineate the importance of a macrophage-collagen fragment axis in physiological SAT expansion. Therapeutic targeting of this process may be a means to prevent pathological adipose tissue remodeling, which in turn may reduce the risk for obesity-related metabolic disorders.

## Significance

Healthy adipose tissue expansion protects against deleterious lipid deposition in “non-adipose tissues” during weight gain. Such adipose tissue expansion relies on effective degradation of collagen structures that surround the growing adipocytes. Here, we found that the rise in degraded collagen during adipose tissue expansion is linked to increased macrophage-mediated collagen uptake. However, macrophages lose this ability in obesity, leading to accumulation of collagen fragments in adipose tissue. These collagen fragments are not just waste products but exert biological actions. For example, we found that they stimulate macrophage proliferation and cause fibroinflammatory effects in fibroblasts. This research suggests that collagen-degrading macrophages and collagen fragments are potential therapeutic targets for the prevention of type-2 diabetes and other conditions of impaired tissue remodeling.

Increased propensity to store excess nutrients in subcutaneous adipose tissue (SAT) protects against deleterious lipid deposition in the visceral compartment. Conversely, visceral adiposity is typically associated with chronic inflammation, insulin resistance, and increased risk for type-2 diabetes and secondary diseases (1). During adipose tissue expansion, the extracellular matrix (ECM) needs to be adaptively degraded to allow for adipocyte growth (hypertrophy), and for new adipocytes to arise from the mesenchymal stem cell niche (hyperplasia) (2). Adipose tissue ECM is mainly composed of fibrillar collagens (types 1, 3, and 5), microfibrillar collagen type 6, and fibronectin, with small presence of laminin (3, 4). While homeostatic collagen turnover is relatively slow, it is accelerated during development, tissue remodeling, and wound healing, and unbalanced in fibrotic and degenerative diseases such as arthritis, atherosclerosis, and cancer (5–7). Dynamic alterations in nutrient availability present a strong signal for the remodeling of adipose tissue that needs to adapt quickly to these changes. Inadequate collagen remodeling during adipose tissue expansion leads to fibrosis and is associated with insulin resistance and increased risk for metabolic diseases (8). Fibrillar collagen degradation in tissues is initiated by extracellular proteolytic cleavage of triple-helical collagen into N-terminal ¾- and C-terminal ¼-fragments (9). This leads to denaturation, exposing epitopes that enable further enzymatic degradation followed by mannose receptor-mediated uptake and lysosomal degradation in fibroblasts and macrophages. In the context of adipose tissue, Matrix Metallopeptidase (MMP)-14 is required for the initial cleavage step and is thus essential for appropriate WAT development and high fat diet (HFD)-induced adipose tissue expansion (10, 11). However, the role of intracellular collagen degradation in the regulation of adipose tissue functionality is largely unknown.

## Results

### Acute 1-wk HFD Challenge Leads to SAT Collagen Type 1 (CT1) Degradation and Accumulation of M2-Like Macrophages.

**Fig. 1.**
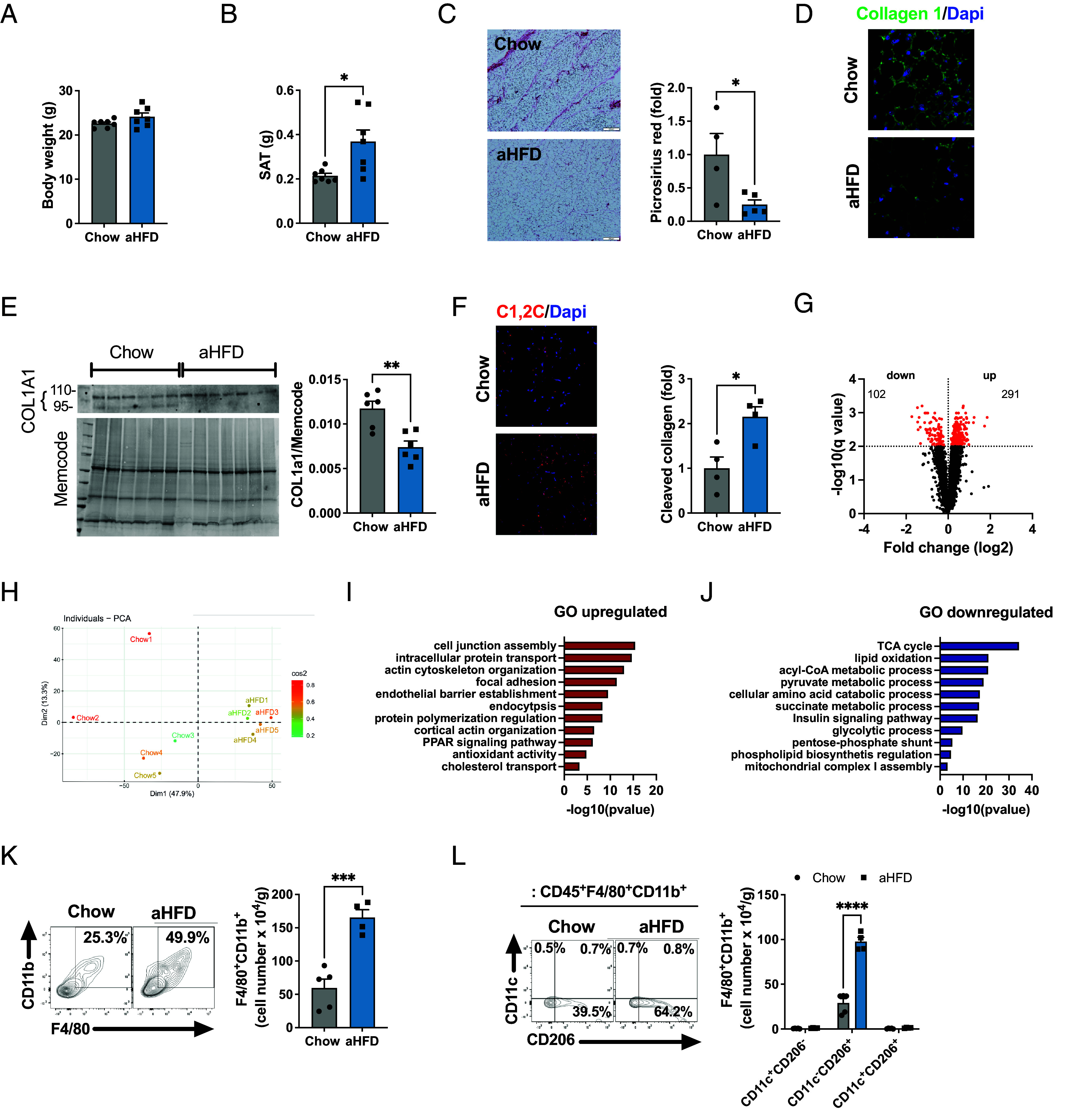
Acute challenge with 1-wk HFD leads to SAT CT1 degradation and accumulation of M2-like macrophages. (*A*) Body and (*B*) SAT weight of 8 wk old male C57BL/6N chow and aHFD mice (n = 7/group). (*C*) Representative image and quantification of picrosirius red staining on SAT of chow (n = 4) and aHFD mice (n = 5). For each animal, images were collected from more than six random fields. Data are presented as fold change compared to chow mice (scale bar, 100 µm). (*D*) Representative image of immunohistofluorescent CT1 staining (green) in SAT of chow and aHFD mice. Nuclei were stained with Dapi (blue, scale bar, 20 µm). (*E*) Western blot analysis of COL1A1 in SAT of chow and aHFD mice (n = 4/group). Band intensity was normalized to total protein amount on the membrane. (*F*) Representative image and quantification of cleaved CT1 in SAT of chow and aHFD mice (n = 4/group). Cleaved CT1 was stained with C1,2C antibody (red) and nuclei were stained with Dapi (blue, scale bar, 20 µm). For each group, tiled (5 x 5) images were taken from at least 3 random fields. Data are presented as fold change compared to control mice. (*G*) Volcano plot and (*H*) PCA of differentially abundant proteins in SAT of chow and aHFD mice (n = 5/group), detected and quantified by LC-MS/MS using TMT multiplexing. Pathway enrichment analysis of (*I*) up-regulated and (*J*) down-regulated proteins in SAT of aHFD mice relative to chow controls. Analysis was done in Cytoscape with GO:Biological process database. (*K*) Representative dot plot and number of macrophages (single live CD45^+^F4/80^+^CD11b^+^) per gram of SAT from chow (n = 5) and aHFD (n = 4) mice. (*L*) Representative dot plot and number of macrophage (single live CD45^+^F4/80^+^CD11b^+^) subsets (M1-like=CD11c^+^CD206^−^, M2-like=CD11c^−^CD206^+^ and mixed M1-M2=CD11c^+^CD206^+^) per gram of SAT from chow (n = 5) and aHFD (n = 4) mice. Data are presented as mean ± SEM. Data are representative of at least three independent experiments (*A*–*F*, *K*, and *L*). Unpaired student’s *t* tests (*A*, *B*, *G*, *I*, *J*, and *K*), 2-way ANOVA with Fisher’s post hoc test (*L*). Multiple unpaired *t* test with Welch correction, corrected with false discovery rate set to 1% cut-off (q value above 2 is significant). *P < 0.05, ***P < 0.001.

To investigate mechanisms of physiological SAT expansion, we challenged 7-wk-old male C57BL/6N mice with 1-wk HFD (in the following referred to as acute HFD, aHFD). Although aHFD showed no effect on body weight (Fig. 1*A*), it led to significant increase in the SAT (Fig. 1*B*) and epidydimal white adipose tissue (EWAT) weights (*SI Appendix*, Fig. S1*A*) compared to control mice on regular chow. Furthermore, aHFD mice were glucose intolerant (*SI Appendix*, Fig. S1*B*), most likely due to acute lipid overload in liver, as judged by increased triglyceride levels (*SI Appendix*, Fig. S1*C*). The SAT expansion in aHFD mice is likely due to adipocyte hypertrophy (12); accordingly, we found a lower percentage of smaller-sized adipocytes and a higher percentage of larger-sized adipocytes in SAT of aHFD mice (*SI Appendix*, Fig. S1*D*). However, the SAT abundance and proliferation of stem cells (Lin^−^Sca1^+^) increased in aHFD mice (*SI Appendix*, Fig. S1 *E* and *F*), presumably allowing for hyperplastic expansion at later stages.

SAT of lean mice has relatively high ECM levels, primarily organized as collagenous “streaks,” mainly composed of CT1 that spread across SAT dividing it into smaller units. In line with previous findings (11, 13), aHFD leads to the disappearance of these streaks with a 50% reduction in CT1 levels (Fig. 1 *C*–*E*), thus facilitating adipocytes expansion. To further confirm collagenolytic activity, we probed SAT tissue sections with an antibody that recognizes the C-terminal neoepitope of the CT1 ¾ fragment (14). While chow-control SAT showed faint immunoreactivity (indicating little to no collagenolytic activity), aHFD expressed marked increase in CT1 fragments (Fig. 1*F*), which is in line with a previous report (11). Other major collagens (6 and 3) remained unchanged upon aHFD challenge (*SI Appendix*, Fig. S1 *G* and *H*). In order to further identify and quantify the proteomic changes of expanding SAT, we employed liquid chromatography-mass spectrometry/mass spectrometry (LC-MS/MS) using tandem mass tag (TMT) labeling. This analysis showed that aHFD-induced expansion changes the proteomic landscape of SAT, with almost 400 differentially expressed proteins compared to chow controls (Fig. 1*G*). Principal component analysis (PCA) showed that the SAT aHFD proteome was distinct from chow controls (Fig. 1*H*). To further analyze the altered protein expression, we performed pathway enrichment analysis using GO:Biological process and Kyoto Encyclopedia of Genes and Genomes ontologies. Focal adhesion and endocytosis were among the most upregulated pathways in aHFD SAT (Fig. 1*I*). This could reflect the ongoing remodeling process where cells need to establish new contacts with the ECM (15). As expected, the aHFD SAT proteome was skewed toward lipid accumulation as evident from significantly up-regulated PPAR signaling pathway and cholesterol transport (Fig. 1*I*). Pathway enrichment analysis of down-regulated proteins further confirmed transition of aHFD SAT toward an energy-storing mode. TCA cycle, lipid oxidation, and mitochondrial respiratory chain complex I assembly pathways were all down-regulated in aHFD SAT compared to chow littermates (Fig. 1*J*). Apart from the secreted protein acidic and cysteine rich that was up-regulated in aHFD proteome, there were no differences in the abundances of other proteins that have been linked to adipose tissue fibrosis/dysfunction (*SI Appendix*, Fig. S1*I*). Additionally, the mRNA expression of ECM-related genes was similar between groups (*SI Appendix*, Fig. S1*J*).

Macrophages play a central role in tissue homeostasis by supporting balanced tissue architecture that can include both tissue growth and tissue loss (16). To test how aHFD affects adipose tissue macrophages, we analyzed the stroma vascular fraction (SVF) of SAT and EWAT with flow cytometry. Remarkably, aHFD challenge provoked significant SAT-macrophage accumulation, as judged by doubled number of live CD45^+^F4/80^+^CD11b^+^ cells per gram of tissue compared to chow controls (Fig. 1*K*). We identified M2-like macrophages (defined as CD11c^−^CD206^+^) (17) as the major expanding subset in aHFD SAT (Fig. 1*L*). This was not observed in EWAT, where the number of macrophages was similar between the groups (*SI Appendix*, Fig. S1*K*). In further support of SAT-restricted macrophage expansion, we found no changes in numbers of macrophages, monocytes, nor lymphocyte subsets (T and B cells) in spleen, a site of systemic immune response (*SI Appendix*, Fig. S1*L*). We detected no changes in the number of SAT neutrophils (*SI Appendix*, Fig. S1*M*). However, mast cells, although being a small population in SAT, expanded upon aHFD challenge (*SI Appendix*, Fig. S1*N*), in line with their proposed role in adipose tissue remodeling (18–20).

In conclusion, aHFD provokes major SAT-remodeling as evident from CT1 degradation, distinct changes in the proteome, and SAT-restricted accumulation of M2-like macrophages.

### Acute HFD Drives Proliferation of SAT Resident Macrophages.

**Fig. 2.**
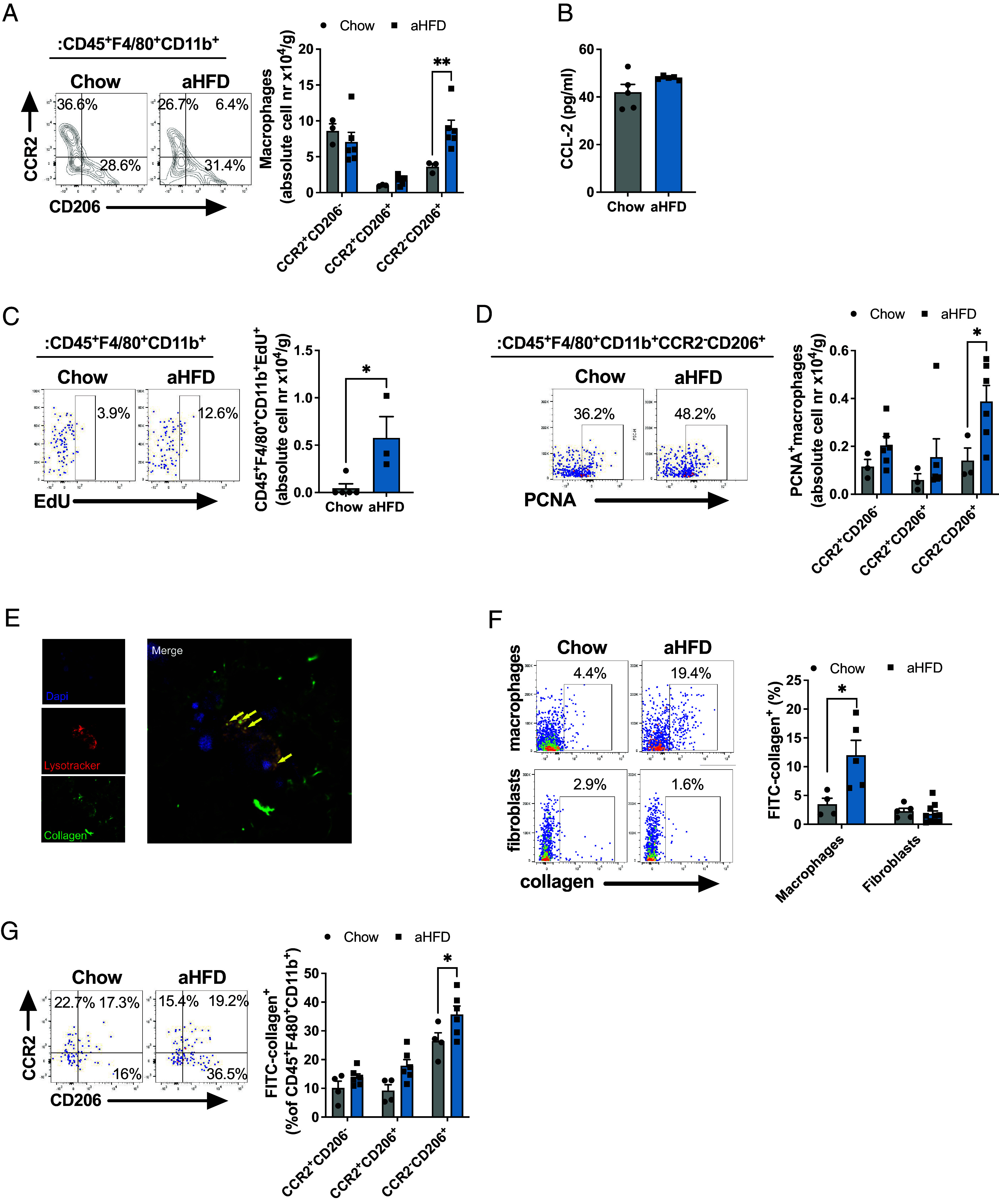
Acute HFD drives proliferation of SAT resident macrophages that engage in collagen endocytosis. (*A*) Representative dot plot and number of infiltrated (CCR2^+^CD206^−^ and CCR2^+^CD206^+^) and resident (CCR2^−^CD206^+^) macrophages (single live CD45^+^F4/80^+^CD11b^+^) per gram of SAT from chow (n = 7, some samples pooled) and aHFD (n = 6) mice. (*B*) Serum levels of CCL-2 from chow (n = 5) and aHFD mice (n = 4). (*C*) Representative dot plot and number of proliferating (EdU^+^) macrophages (single live CD45^+^F4/80^+^CD11b^+^) per gram of SAT from chow (n = 5) and aHFD (n = 4) mice. (*D*) Representative dot plot and number of PCNA^+^ total (single live CD45^+^F4/80^+^CD11b^+^), infiltrated (CCR2^+^CD206^−^ and CCR2^+^CD206^+^ of total), and resident (CCR2^−^CD206^+^ of total) macrophages per gram of SAT of chow (n = 7, some samples pooled) and aHFD (n = 7) mice. (*E*) Representative image of collagen-endocytosing macrophages. Ex vivo sorted macrophages (F4/80^+^ SVF of SAT) where immersed in neutralized FITC-collagen and left to polymerize. Collagen (green) forms the fibers similar to in vivo settings. Lysosomes were stained with Lysotracker red (red). Nuclei were stained with Dapi (blue). Macrophages endocytose collagen which can be noted as co-localization of green (collagen) and red (lysosomes) signal. (*F*) Collagen endocytosis assay. Frequencies of FITC-collagen^+^ macrophages (single live CD45^+^F4/80^+^CD11b^+^) and fibroblasts (single live CD45^-^PDGRFα^+^) from SAT of chow and aHFD mice (n = 10/group). Cells were magnetically sorted (macrophages: F4/80^+^ of SVF, fibroblasts: F4/80^−^CD45^−^CD90.2^+^ of SVF). (*G*) Collagen endocytosis assay- subset distribution of collagen endocytosing macrophages. Frequencies of FITC-collagen^+^ infiltrated (CCR2^+^CD206^−^ and CCR2^+^CD206^+^) and resident (CCR2^−^CD206^+^) macrophages (single live CD45^+^F4/80^+^CD11b^+^). Data are presented as mean ± SEM and are representative of two or more independent experiments. Unpaired student’s *t* tests (*B*, *C*, and *F*), two-way ANOVA with Fisher’s post hoc test (*A*, *D*, and *G*). *P < 0.05, **P < 0.01

HFD-induced obesity is typically associated with increased recruitment of macrophages from the blood monocyte niche to EWAT and, albeit to less extent, SAT (21). To test whether observed SAT macrophage accumulation upon aHFD challenge is a consequence of increased monocyte recruitment, we analyzed SAT macrophages for the expression of CCR2, a chemokine receptor expressed on macrophages originating from classical monocytes (22–25). We noted no difference in CCR2^+^ macrophage subsets between the groups, but the CCR2^−^CD206^+^ macrophage subset significantly expanded upon aHFD (Fig. 2*A*). Furthermore, levels of CCL2, a chemokine considered as a major driving signal for monocyte recruitment in pathological states (26), were unchanged both with respect to circulating protein level (Fig. 2*B*) and gene expression (27). Overall, this indicates that the accumulation of macrophages upon aHFD challenge is not driven by infiltration from the blood monocyte pool. Rather, it could result from local proliferation of resident macrophages. Indeed, the density of EdU^+^ macrophages was significantly higher in SAT of aHFD mice compared to chow controls (Fig. 2*C*). A similar increase was observed when we analyzed macrophages for the prolif maeration marker Proliferating Cell Nuclear Antigen (PCNA; Fig. 2*D*). Notably, the expression of PCNA among the different macrophage subsets was increased only in the CCR2^−^CD206^+^ group in aHFD SAT (Fig. 2*D*), suggesting selective proliferation of residentcrophages. Increased proliferation was not due to elevated levels of IL-4 or M-CSF, as we detected no changes in protein amount between the groups (*SI Appendix*, Fig. S2 *A* and *B*); and we saw no difference in proliferation of SAT fibroblasts, EWAT macrophages, or blood monocyte subsets between the groups (*SI Appendix*, Fig. S2 *C*–*E*). Thus, aHFD challenge provokes expansion of SAT-macrophages through local proliferation of resident M2-like macrophages rather than through monocyte infiltration.

### Acute HFD Increases the Engagement of M2-Like Macrophages in CT1 Endocytosis.

**Fig. 3.**
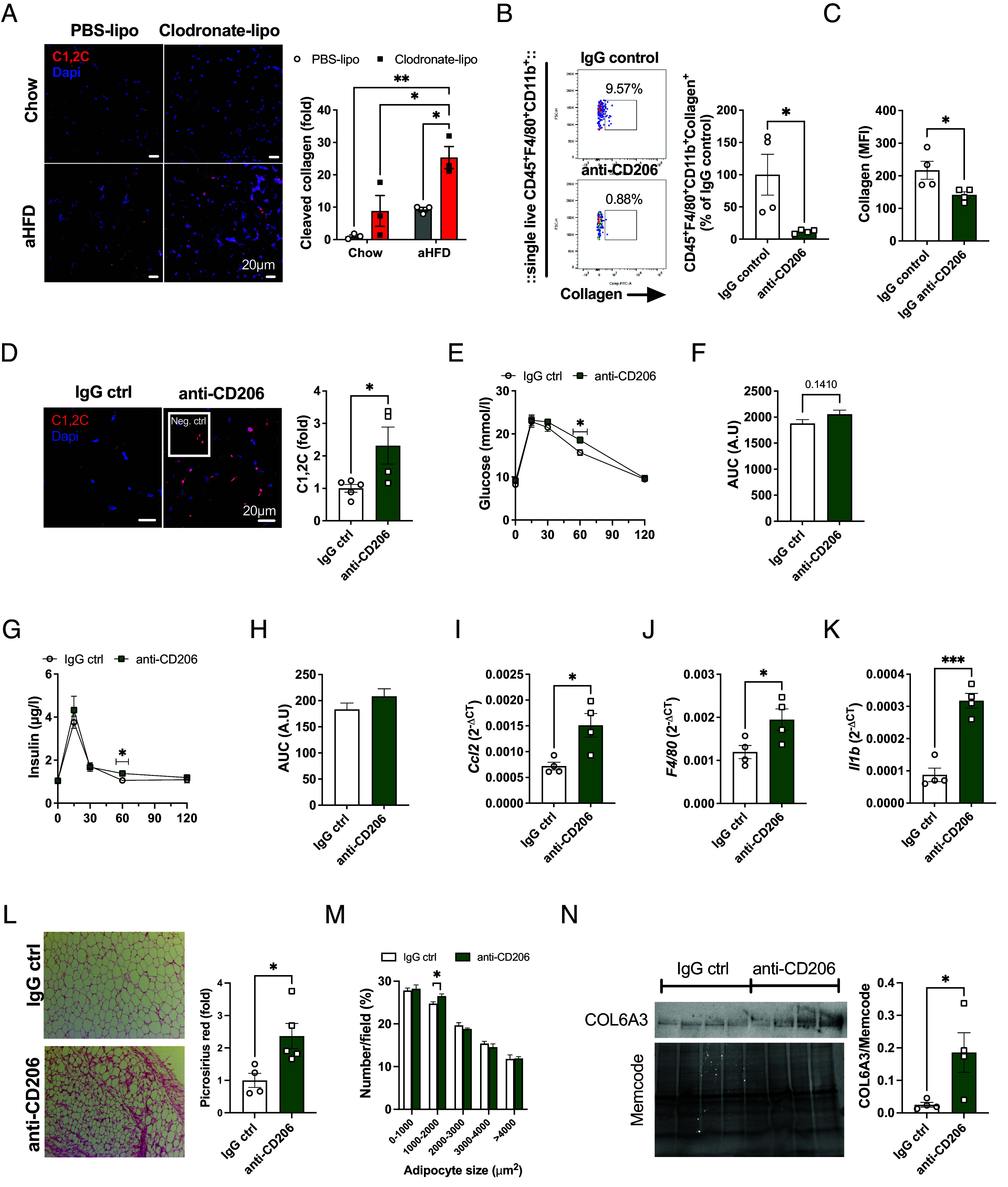
CT1 endocytosis in SAT macrophages is mediated by CD206 receptor. (*A*) Representative images and quantification of C1,2C staining in SAT. Chow and aHFD mice received Clodronate-liposomes (40 mg/kg bw) into one SAT fat pad and control PBS-liposomes into another fat pad on day 0 and day 3 of aHFD course (n = 3/group). Cleaved CT1 was stained with C1, 2C antibody (red) and nuclei were stained with Dapi (blue, scale bar, 20 µm). For each group, tiled (5 x 5) images were taken from at least three random fields. Data are presented as fold change compared to chow PBS SAT. (*B* and *C*) Representative dot plots and quantification of ex vivo collagen endocytosis assay in the presence of neutralizing anti-CD206 antibody or IgG control (n = 4/group). SAT-macrophages from lean mice were magnetically sorted (F4/80^+^) and treated with FITC-collagen (15 µg/mL) in the presence of anti-CD206 antibody or IgG control (10 µg/mL). Collagen^+^ macrophages as (*B*) % of total macrophages and (*C*) collagen uptake per cell, measured as MFI values. (*D*–*N*) In vivo antibody-mediated CD206 neutralization. Mice were treated with anti-CD206 or IgG control (1 mg/kg) antibody every other day during the aHFD-challenge. (*D*) Representative images and quantification of C1,2C (red) staining in SAT of IgG- (n = 4) or anti-CD206-treated (n = 5) aHFD mice. Nuclei were stained with Dapi (blue, scale bar, 20 µm). Data are presented as a fold change of IgG control. (*E*) Blood glucose levels and (*F*) area under the curve (AUC) during oral glucose tolerance test (OGTT, n = 5/group). (*G*) Serum insulin levels and (*H*) AUC during OGTT (n = 5/group). mRNA expression of (*I*) *Ccl2* (*J*) *F4/80,* and (*K*) *Il1* in SAT of anti-CD206 or IgG-treated mice (n = 4/group). Expression is relative to *Bactin.* (*L*) Representative images and quantification of picrosirius red staining in SAT of anti-CD206 or IgG-treated mice (n = 4 to 5). For each sample, images were collected from more than six random fields. Data are presented as fold change compared to IgG control SAT (scale bar, 100 µm). (*M*) Adipocyte size distribution and (*N*) average adipocyte size calculated from the picrosirius red staining with ImageJ software and Adiposoft plugin. (*O*) Western blot analysis of COL6A3 in SAT of anti-CD206 or IgG-treated mice (n = 4/group). Band intensity was normalized to total protein amount on the membrane. Data are presented as mean ± SEM and are representative of two independent experiments (*A*–*C*). Unpaired student’s *t* tests, two-way ANOVA (*A* and *M*). *P < 0.05, **P < 0.01

Based on these observations, we hypothesized that the proliferating M2-like macrophages engage in endocytosis of degraded collagen in expanding SAT. To test this hypothesis, we analyzed the capacity for collagen uptake in SAT-macrophages isolated from chow and aHFD-challenged mice ex vivo. SAT-macrophages were immersed into neutralized fluorescein isothiocyanate (FITC)-labeled CT1. This allowed for the polymerization of collagen with cells inside, thus forming a 3D model that resembles the in vivo setting. After 24 h, we analyzed cells for collagen uptake. Imaging with confocal microscopy showed that collagen formed fibrils in a similar manner as in vivo (Fig. 2*E*). Furthermore, macrophages clearly endocytosed collagen, as apparent from co-localization of collagen (green) and lysosomes (stained red with lysotracker dye) (Fig. 2*E*). To quantify collagen endocytosis, we performed flow cytometry analysis of SAT-sorted macrophages after overnight treatment with FITC-collagen. This analysis showed about a threefold increase in collagen-positive macrophages isolated from SAT of aHFD-challenged mice compared to chow controls (Fig. 2*F*). This was mostly due to enhanced collagen endocytosis in the CCR2^−^CD206^+^ macrophages (Fig. 2*G*). Notably, increased endocytosis was confined to macrophages, as we noted no changes in collagen uptake by fibroblasts (defined as CD45^−^CD90.2^+^PDGRFα^+^ cells), another cell type involved in collagen internalization (28) (Fig. 2*F*). To further demonstrate the role of macrophages in the removal of degraded collagen during ECM-remodeling, we depleted SAT-macrophages with local injection of clodronate-loaded liposomes (*SI Appendix*, Fig. S3*A*). Clodronate induces apoptosis of monocytes/macrophages (29). Successful depletion was confirmed by a marked decrease in *F4/80* mRNA levels in the clodronate-treated fat pads of chow and aHFD mice (*SI Appendix*, Fig. S3*B*), consistent with previous studies (30). Macrophage depletion did not affect the amount of CT1 fragments in chow-fed control animals. However, macrophage depletion in combination with aHFD challenge provoked a striking accumulation of CT1 fragments (Fig. 3*A*) compared to all the other groups, thus confirming that macrophages are critical for the removal of degraded CT1. Furthermore, macrophage depletion during aHFD resulted in increased amounts of pericellular collagen in clodronate-treated aHFD mice (*SI Appendix*, Fig. S3*C*) and elevated mRNA expression of fibrosis and inflammation markers (*SI Appendix*, Fig. S3*D*).

In conclusion, aHFD SAT displays increased collagen endocytosis driven by resident M2-like macrophages.

### CT1 Endocytosis in SAT Macrophages Is Mediated by the CD206 Receptor.

Cellular uptake of collagen is governed by mannose receptors (MRC1/CD206 and MRC2/uPARAP) that show differential cell distribution; CD206 is mostly expressed on macrophages, and MRC2 on fibroblasts (28, 31). To further understand the mechanism of collagen internalization in macrophages, we analyzed whether collagen endocytosis of SAT-sorted macrophages is affected by antibody-mediated CD206 neutralization ex vivo. Treatment with CD206 antibody dramatically reduced the percentage of collagen-positive cells, as well as collagen uptake (Fig. 3 *B* and *C*) implying that CD206 is the main receptor for collagen endocytosis in SAT-macrophages in our setting. To probe for physiological significance of CD206 during SAT-remodeling process, we treated mice with CD206 antibody during aHFD challenge. As expected, antibody-mediated CD206 neutralization augmented the accumulation of degraded collagen in SAT (Fig. 3*D*) while SAT weights were similar between the groups. The anti-CD206-treated aHFD-challenged mice displayed mild metabolic dysfunction as judged from slightly elevated glucose and insulin levels at 60 min following an oral glucose load (Fig. 3 *E*–*H*). Furthermore, anti-CD206 treatment was associated with up-regulated SAT mRNA levels of *F4/80*, *Ccl2,* and *Il1b* (Fig. 3 *I*–*K*) suggesting increased macrophage infiltration. In addition, SAT of anti-CD206-treated mice displayed increased peri-cellular fibrosis (Fig. 3*L*), echoed by a higher percentage of relatively small adipocytes (Fig. 3*M*). In further support of a fibrotic phenotype, protein levels of COL6A3 were elevated in anti-CD206-treated mice (Fig. 3*N*). These data suggest that CD206-mediated collagen endocytosis is essential for physiological SAT expansion during aHFD.

### The Macrophage-Collagen Fragment Axis Is Disrupted in Obese SAT.

**Fig. 4.**
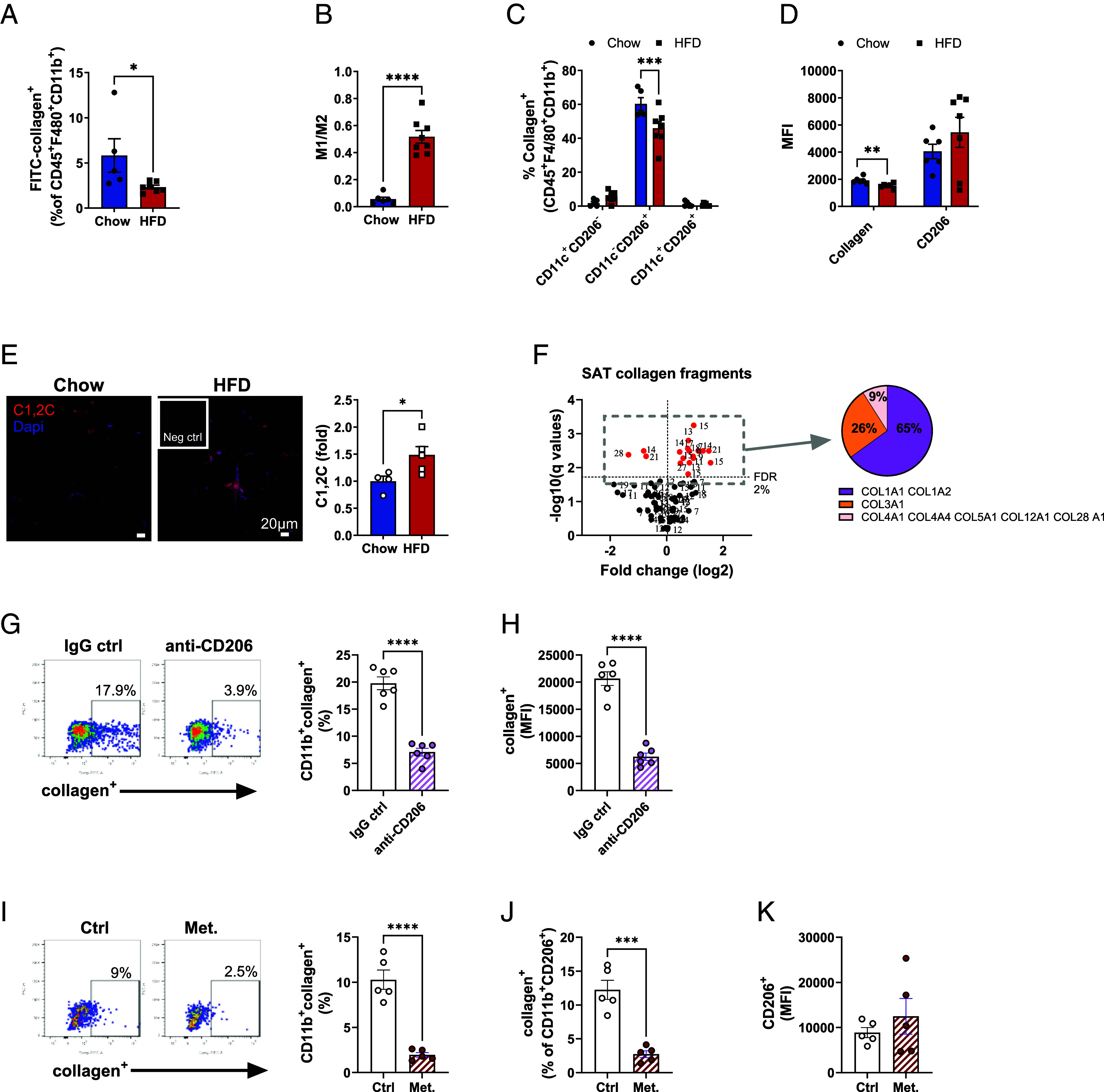
Macrophage-collagen axis is disrupted in SAT of obese insulin resistant mice. (*A*–*F*) Male C57BL/6 mice were placed on chow or HFD for 14 wk. (*A*) Percentage of collagen^+^ macrophages (single live CD45^+^F4/80^+^CD11b^+^) magnetically sorted from SAT of chow (n = 6) or mice fed with HFD (n = 8). (*B*) M1/M2 ratio in SAT of chow and HFD mice. M1 macrophages were defined as CD11c^+^CD206^−^ and M2 as CD11c^−^CD206^+^ of total macrophages (single live CD45^+^F4/80^+^CD11b^+^). (*C*) Subset distribution of collagen^+^ macrophages. (*D*) Mean fluorescence intensity of collagen^+^ and CD206^+^ macrophages. (*E*) Representative images and quantification of C1,2C staining (red) in SAT of chow (n = 4) and HFD (n = 5) mice. Nuclei were stained with Dapi and data are presented as fold change of chow controls (scale bar, 20 µm). (*F*) Volcano plot of various fragmented collagens found in SAT of chow and HFD mice (n = 5/group). The number indicates amino acid length of each fragment (sequences are shown in *SI Appendix*, Table S1). Collagen^+^ human PBMC-derived M2-like macrophages (live CD3^−^CD19^−^CD56^−^CD66b^−^CD1c^−^CD11b^+^CD206^+^) in the presence of (*G*–*H*) anti-CD206 antibody or IgG control (10 µg/mL both) or (*I*–*J*) in the presence or absence of metabolic cocktail (25 mM glucose, 0.5 mM palmitate-BSA, 10 nM insulin). MFI values of (*H*) collagen and (*K*) CD206. Data are presented as mean ± SEM. Unpaired student’s *t* tests, two-way ANOVA (*C*), and multiple unpaired *t* test with Welch correction, corrected with false discovery rate set to 2% cut-off (*F*). *P < 0.05, **P < 0.01, ***P < 0.001, ****P < 0.0001

A recent study shows that circulating CT1 fragments (C1M) are linked to both obesity and a more severe disease phenotype in patients with asthma (32), suggesting imbalance between extracellular and intracellular collagen degradation. To test whether the macrophage-CT1 fragment axis in SAT is disturbed in metabolically challenged conditions, we compared the collagen endocytosis capacity between SAT macrophages from lean and HFD-induced obese insulin-resistant male mice. As expected, the engagement in collagen endocytosis was diminished in the obese setting (Fig. 4*A*). This reduction can be a consequence of phenotypic switch toward pro-inflammatory M1 macrophages that, by default, have a lower capacity for endocytosis [Fig. 4*B*; (33)]. However, we noted that reduced CT1 endocytosis in HFD macrophages was confined to the M2-like subset, that displayed about 25% reduction of collagen^+^ cells in SAT of obese mice (Fig. 4*C*). This was echoed by impaired collagen uptake per cell in obese M2-like macrophages, as judged by decreased collagen MFI (mean fluorescent intensity) values compared to chow controls (Fig. 4*D*). This difference was not due to decreased expression of CD206 receptor, as CD206 MFI values were similar between the groups (Fig. 4*D*). In accordance, we found 1.5-fold elevated levels of degraded CT1 in SAT of HFD-induced obese insulin-resistant mice compared to chow controls (Fig. 4*E*). This difference is even more prominent if the larger adipocyte size in obese mice is considered. Together, our data indicate impaired macrophage clearance of fragmented collagen in obese SAT. We further argued that increased MMP levels in obese adipose tissue (34) may lead to more excessive extracellular degradation and increased abundance of shorter fragments. To test this assumption, we analyzed SAT collagen fragment composition and relative abundances using LC-MS/MS with TMT multiplexing on protein lysates depleted of larger proteins (>30 kDa). This analysis detected 72 different collagen fragments, of which 16 were more and 3 less abundant in obese SAT (Fig. 4*F* and Dataset S1). All but five of the detected collagen fragments contained Gly-X-Y repeats and can thus be defined as collagenous peptides. They also included at least one hydroxyproline that is a hallmark of mature ECM collagens (35). Furthermore, 65% of the detected fragments come either from COL1A1 or COL1A2, 26% come from COL3A1, and the remaining 9% are from COL4A1, COL4A4, COL5A1, COL12A1, and COL28A1 (Fig. 4*F*). In line with our assumption, the SAT collagen fragments did not only increase in abundance but also shifted in size and composition: 13 of the up-regulated fragments in obese SAT were <18 amino acids long, whereas only one of the down-regulated fragments was as short; this fragment was also very similar in sequence to two of the up-regulated fragments (Dataset S1). The collagen fragment length may be of importance as collagenous peptides have an inherent ability to form triple helices, but only if they are at least 18 amino acids long (36). Thus, shorter fragments expose epitopes that normally are hidden within the triple-helical structure.

To test whether our findings can be applied to a human setting, we measured collagen endocytosis in human PBMC-derived M2-like macrophages. Indeed, CD206 is the main collagen endocytosing receptor also in human macrophages as judged by the pronounced reduction in collagen endocytosis upon antibody-mediated CD206 neutralization (Fig. 4 *G* and *H*). Similarly to SAT macrophages of obese mice, challenge with obesogenic conditions (palmitate, high glucose, and high insulin) reduced the engagement in collagen endocytosis in the human M2-like macrophages (Fig. 4*I*). This effect was seen even within the CD206^+^ compartment in which the surface CD206 expression was similar (Fig. 4 *J* and *K*), implying that the reduction in collagen endocytosis was not solely due to a phenotype switch.

Collectively, these data demonstrate a disturbed macrophage-collagen fragment axis with impaired endocytosis and elevated levels of short collagen fragments in obese SAT.

### Collagen Fragments Induce Inflammation and Fibrosis in Fibroblasts and Adipocytes, but Proliferation in M2-Like Macrophages.

**Fig. 5.**
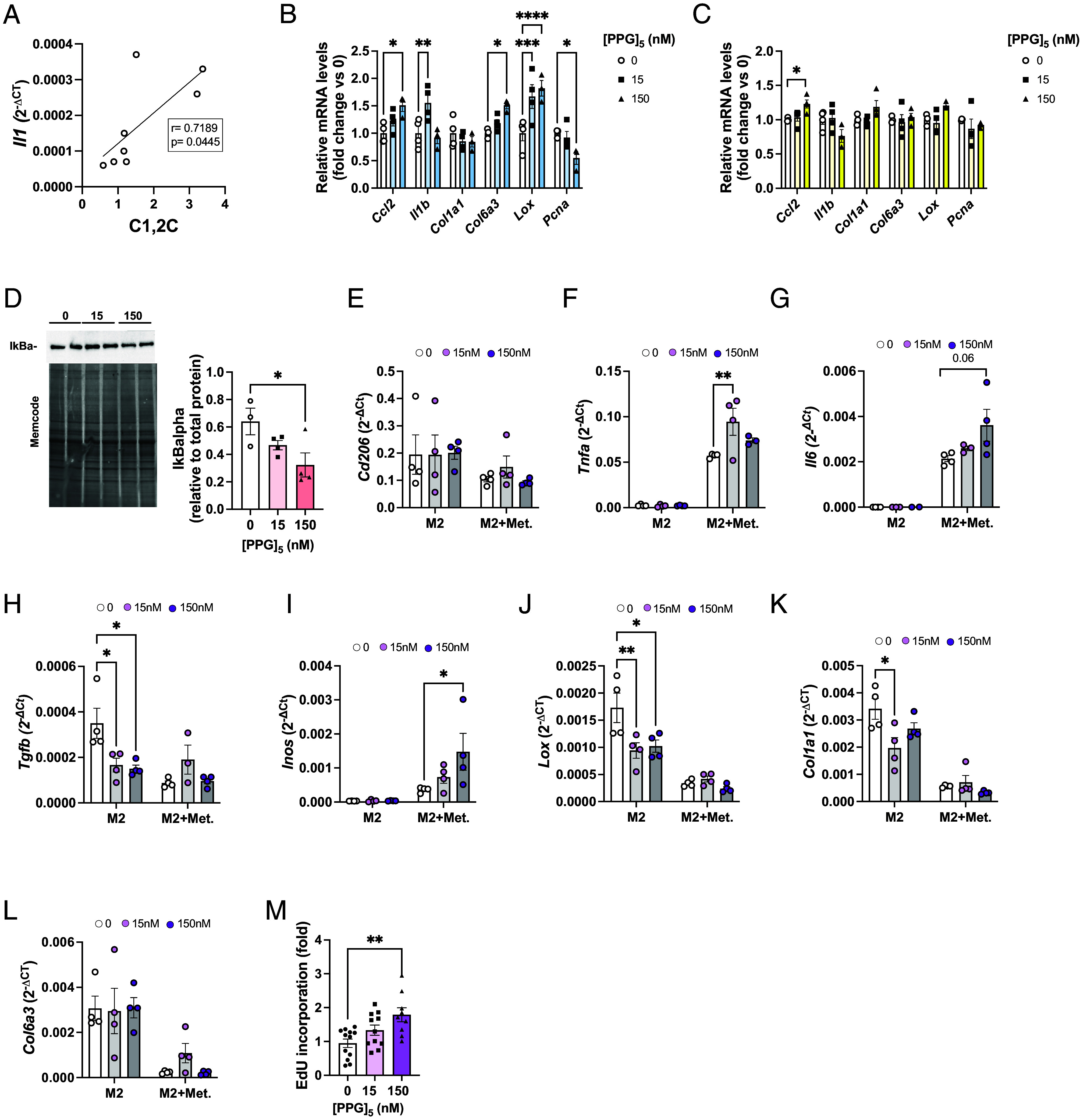
Collagen fragments induce inflammation and fibrosis in pre- and adipocytes, but proliferation in M2-like macrophages. (*A*) Pearson correlation of mRNA expression of Il1 and the amount of collagen fragments in the SAT of mice treated with anti-CD206 or IgG control. mRNA expression (relative to untreated cells) after in vitro treatment of (*B*) 3T3-L1 fibroblasts and (*C*) differentiated 3T3-L1 adipocytes. (*D*) Western blot analysis of IkBalpha in 3T3-L1 pre-adipocytes after 24 h treatment with [PPG]_5_. Band intensity was normalized to total protein amount on the membrane. (*E*–*L*) mRNA expression in M2 BMD macrophages after 24 h treatment with [PPG]_5_ (n = 3 to 4 per group) in the presence or absence of metabolic cocktail (25 mM glucose, 0.5 mM palmitate-BSA, 10 nM insulin). Expression is relative to *Bactin.* (*M*) EdU incorporation in M2 BMD macrophages after 24 h treatment with collagen mimetic peptide (15 and 150 nM, n = 9 to 12/group). Data are presented as mean ± SEM and are representative of three independent experiments (*A*–*D*). One significant outlier value was detected with Grubb’s outlier test (*P* < 0.05) and removed (*F* and *G*). One-way (*B*–*D* and *M*) and two-way (*E*–*L*) ANOVA. *P < 0.05, **P < 0.01.

Our aHFD challenge and HFD-induced obesity results suggest that extracellular collagen degradation pathways cannot fully compensate for impaired macrophage-mediated collagen degradation. Moreover, we also noted that the SAT levels of CT1 fragments positively correlated with *Il1b* (Fig. 5*A*). This prompted us to test whether CT1 fragments are active regulators of tissue remodeling processes. To this end, we used [proline-proline-glycine]_5_ ([PPG]_5_), a peptide that mimics most of the upregulated collagen fragments in obese SAT. Low concentrations of [PPG]_5_ (15.6 and 62.5 nM) for 24 h had a small positive effect on viability in 3T3-L1 fibroblasts, while higher concentrations were without effect (*SI Appendix*, Fig. S4 *A* and *B*). Based on this and published literature (37), we used concentrations of 15 and 150 nM. Twenty-four hours treatment of 3T3-L1 fibroblasts with high (150 nM) concentration of [PPG]_5_ led to increased mRNA expression of *Ccl2*, *Col6a3*, and *Lox* (Fig. 5*B*), while the expression of *Pcna* was decreased and *Col1a1* was unchanged (Fig. 5*B*). Treatment with the lower concentration (15 nM) of [PPG]_5_ increased the *Il1b* and *Lox* levels, but was without effect on *Col1a1, Col6a3, Ccl2,* and *Pcna* (Fig. 5*B*). Similar, though less potent effect was seen in 3T3-L1 adipocytes; collagen mimetic peptide at 150 nM up-regulated the expression of *Ccl2* (Fig. 5*C*), with trends toward increased *Col1a1* and *Lox* while the expression of the other genes was unaffected (Fig. 5*C*). Treatment with the lower concentration (15 nM) of [PPG]_5_ had no effect on the expression of these selected genes in 3T3-L1 adipocytes (Fig. 5*C*). Mechanistically, the observed effects of [PPG]_5_ are at least in part due to activation of Nuclear factor kappa-light-chain-enhancer of activated B cells (NF-κB) signaling pathway, as 24 h treatment of 3T3-L1 fibroblasts with [PPG]_5_ dose-dependently induced degradation of the NF-κB inhibitor- nuclear factor of kappa light polypeptide gene enhancer in B-cells inhibitor, alpha (IκB alpha) (38) (Fig. 5*D*).

The dose-dependent effects of [PPG]_5_ could reflect the ability of cells to efficiently internalize fragments via receptors such as CD206 or MRC2 (39). In support of this, [PPG]_5_ treatment of M2-like bone marrow-derived macrophages (BMDMs) markedly down-regulated the expression of *Tgfb*, *Lox,* and *Col1a1*, suggesting anti-fibrotic effect of fragmented collagen on this cell type (Fig. 5 *E*–*L*). Furthermore, [PPG]_5_ dose-dependently induced proliferation of M2-like BMDM macrophages, as judged by increased EdU incorporation after 24 h treatment (Fig. 5*M*). In agreement with previous reports (40), metabolic activation of M2-like macrophages with high glucose, insulin, and palmitate increased expression of *Tnfa*, *Inos,* and *Il6*, without effect on *Cd206* expression (Fig. 5 *E*–*G*, *I*). This pro-inflammatory effect was further potentiated with [PPG]_5_ treatment (Fig. 5 *F*–*G*, *I*), suggesting that metabolically activated macrophages are less efficient in the removal of fragmented collagen, thus allowing for its proinflammatory actions.

Collectively, we demonstrate that collagenous fragments can be actively involved in tissue remodeling processes via their fibroinflammatory and proliferative effects.

## Discussion

We show that aHFD-induced SAT expansion provokes CT1 degradation, paralleled by proliferation of resident M2-like macrophages. These SAT macrophages are essential for the intracellular degradation of CT1 fragments, a function that is impaired in obesity associated with increased abundance of relatively short collagen fragments. Collagen endocytosis in SAT macrophages is governed by CD206, and antibody-mediated inhibition of CD206 leads to maladaptive SAT expansion with increased markers of inflammation and fibrosis. We also demonstrate that short collagen fragments dose-dependently induce pro-inflammatory and pro-fibrotic changes in 3T3-L1 pre- and adipocytes and metabolically activated bone marrow-derived M2-like macrophages. Thus, chronically increased levels of fragmented collagen provide a mechanistic link between reduced collagen endocytosis in M2-like macrophages and adipose tissue dysfunction in obesity.

Adipose tissue-resident macrophages originate mostly from yolk sac erythro-myeloid progenitors (41). These macrophages are involved in sustaining tissue homeostasis and are maintained by self-renewal or monocyte replenishment (41). We show that SAT resident M2-like macrophages proliferate upon aHFD challenge and take part in CD206-mediated collagen endocytosis. M2-like macrophages have previously been reported to have a high endocytic capacity, ranging from 80 to 100% compared to ~20% of the M1-like macrophages (33). Thus, M2-like macrophage proliferation may accelerate the removal of degraded CT1 in expanding SAT. Notably, weight loss is associated with accumulation of M2-like macrophages (42, 43). Moreover, several clinical studies suggest the importance of macrophages in adipose tissue collagen remodeling. For instance, SAT collagen is positively associated with M2-like macrophages before bariatric surgery but negatively after (44) and adipose tissue macrophages in insulin-resistant subjects are linked to increased collagen six levels and fibrosis (45). While local proliferation of resident macrophages in obesity may contribute to metabolic disturbances and insulin resistance (46, 47), we suggest that the aHFD-induced expansion of resident macrophages represents an adaptive response to ensure appropriate tissue remodeling. In support of this notion, clodronate-mediated depletion of macrophages in aHFD-challenged mice leads to maladaptive SAT remodeling, with increased markers of fibrosis and inflammation. Our data are thus in line with a study that demonstrates elevated markers of inflammation in clodronate-treated HFD-fed mice (48).

One possible stimulus for the observed aHFD-induced SAT resident macrophage proliferation could be the increased levels of fragmented collagen. In addition, macrophage-mediated efferocytosis of dead neutrophils may contribute (49); neutrophils transiently infiltrate intra-abdominal fat 3 d after HFD feeding (50). Another possibility is that macrophage proliferation is induced by increased mechanical forces in SAT at the beginning of the HFD, similar to post-MI heart healing (51).

In certain cancer types, M2-like macrophages and cancer-associated fibroblasts are the main cell types involved in collagen internalization (52). While SAT fibroblasts endocytosed CT1 in our study, aHFD did not affect their capacity for endocytosis, suggesting that M2-like macrophages are the predominant collagen endocytosing cells in expanding SAT. In agreement with previous studies on bone marrow-derived and peritoneal macrophages (53, 54), we identify CD206 as the dominant receptor involved in macrophage-mediated collagen internalization during SAT expansion: Treatment with anti-CD206 antibody dramatically reduced collagen endocytosis rate in vitro and increased SAT CT1-fragment accumulation in vivo. Furthermore, we found that CCR2^−^CD206^+^ macrophages engaged more in CT1 endocytosis than CCR2^+^macrophages. Similar results have been reported in a model of early cardiac hypertrophy where resident macrophages were shown to inhibit fibrosis while the recruited monocyte-derived CCR2^+^ macrophages had the opposite effect (55). This collagen degrading function has primarily been attributed to CCR2^+^CD206^+^ macrophages (56, 57). However, these studies focus on wound healing and tumors that typically display higher infiltration of monocyte-derived macrophages than observed in aHFD-challenged SAT.

Interestingly, antibody-mediated inhibition of CD206 during aHFD provoked changes that are similar to obese insulin-resistant conditions with reduced glucose tolerance and increased markers of inflammation/macrophage infiltration and fibrosis. We acknowledge that the difference in glucose tolerance between groups is rather modest. But early impairments in glucose tolerance in HFD-fed mice are thought to result primarily from the acute tissue lipid overload, especially in the liver (58, 59). Our results therefore imply that physiological SAT expansion and CD206-mediated collagen endocytosis provide protection against systemic metabolic disturbances in response to acute lipid/caloric overload. It is, however, possible that systemic CD206 neutralization during aHFD challenge causes effects that are unrelated to collagen endocytosis in SAT.

In HFD-induced obese insulin-resistant conditions, we observe that M2-like SAT macrophages are much less engaged in collagen endocytosis, associated with increased levels of fragmented collagen. Furthermore, most collagen fragments that were enriched in obese SAT were short (less than 18 amino acids) while two of the three down-regulated fragments were longer than 18 amino acids. This difference in SAT fragment levels and composition could reflect both decreased intracellular degradation (this study) and increased proteolytic activity in obesity (34, 60). The lowered capacity for collagen endocytosis in M2-like macrophages is not due to loss of CD206 surface expression. This suggests that M2-like macrophages are occupied with other tasks and/or have lost their functionality in metabolically disturbed adipose tissue. Indeed, CD206^+^ macrophages often play a pro-fibrotic role in pathophysiological settings (61, 62) as opposed to the anti-fibrotic role in aHFD SAT expansion (this study) and in a model of early cardiac hypertrophy response (55). In obese, insulin-resistant adipose tissue, CD9^+^ macrophages, that also express higher levels of CD206 and CD16, accumulate around dying adipocytes forming “crown-like structures” (63). Engulfment of dead cells, or efferocytosis, is associated with a “satiated” phenotype, characteristic of reduced endocytic capacity, increased TGF-beta secretion, and immune unresponsiveness (64). It is thus tempting to speculate that macrophages acquire a satiated phenotype leading to reduced endocytic activity in obese SAT.

Our findings are in accordance with recent research showing that impaired intracellular degradation is linked to aggravated fibroinflammatory changes in obese adipose tissue and in bleomycin-induced lung injury (65–67). It seems possible that impaired macrophage-mediated intracellular degradation leads to increased release of prolidase that in turn can trigger fibroinflammatory actions via EGFR-signaling (65). In addition to this mechanism, the short collagen peptides that accumulate in obese SAT exert fibroinflammatory effects. Notably, effects of collagen mimetic peptide treatment in cultured 3T3-L1 fibroblasts and adipocytes and metabolically challenged BMDMs mimimicked the main findings of our anti-CD206 in vivo study and are also in line with observations in obese/insulin resistant settings. While we cannot exclude additional effects of CD206 neutralization on macrophage and SAT functionality, our in vitro data indicate that some of the maladaptive changes in anti-CD206-treated and obese SAT can be provoked by direct effect of collagen fragments, though to what extent is yet to be elucidated. For instance, 24 h treatment with high concentration of the short collagen mimetic peptide [PPG]_5_ increased the expression of chemokine *Ccl2* in vitro, a finding that is in line with previous research; gelatin (non-fibrillar denatured collagen), fragmented fibrillar collagen, short collagen mimetic peptides (e.g., [PPG]_5_/[P-hydroxyproline-G]_5_), and PGP all are exert chemotactic activity (68–70). We also demonstrate that [PPG]_5_ exerts pro-fibrotic effects as judged by increased expression of *Col6a3* and *Lox*. Mature collagen-6 microfibrils facilitate adipogenesis (71), while C-terminal cleavage product of COL6A3, endotrophin, promotes adipose tissue fibrosis (72). We therefore postulate that COL6A3 plays both an adaptive and maladaptive role during SAT expansion promoting healthy hyperplastic growth but also excessive collagen accumulation. In contrast to effects observed in 3T3-L1 fibroblasts, [PPG]_5_ treatment reduced the *Tgfb*, *Lox,* and *Col1a1* levels in unchallenged M2-like BMDMs suggesting that [PPG]_5_ and/or collagen endocytosis trigger an anti-fibrotic negative feedback response that is missing in metabolically challenged conditions. However, the mechanism(s) for the tissue remodeling actions of fragmented collagen are yet largely unknown. Here, we provide evidence that [PPG]_5_ induces the NF-κB-signaling pathway, the main regulator of inflammatory processes (73, 74), which could be one of the mechanisms for fragment-induced fibroinflammatory changes. Another possibility is that the PGP epitope (present in many of the up-regulated fragments) and CXCR2 are involved (69, 75, 76).

Limitations of this study are that naturally occurring fragments may show different and/or additional effects than the collagen mimetic peptide that we used and that we solely used male mice. Detailed studies of the actions of naturally occurring fragments, more thorough characterization of the collagen degrading CD206^+^ macrophage population including possible sex differences are part of our ongoing efforts.

In conclusion, this study highlights the importance of collagen-degrading macrophages and efficient removal of collagen fragments in adaptive, weight gain–induced adipose tissue remodeling. Our data suggest that impaired macrophage-mediated intracellular collagen degradation in obese SAT cannot be fully compensated for by extracellular collagen degradation. We conclude that collagen fragments, rather than being inert metabolites and solely markers of tissue remodeling, actively participate in shaping the SAT microenvironment. Further research in this area may identify novel targets in the prevention of type-2 diabetes in subjects with obesity and in other areas of impaired tissue remodeling.

## Materials and Methods

### Mouse Studies.

All experimental protocols for mouse studies were approved by Animal Ethics Committee at the Administrative Court of Appeals in Gothenburg, Sweden. Six-week-old C57BL/6N wild-type mice were randomly assigned to groups and fed either standard chow or high-fat diet (HFD, 60% fat, 20% protein, and 20% carbohydrate, D12492, Research Diets Inc., New Brunswick, NJ, USA) for 1 wk (acute HFD) or 14 wk, after which they were sacrificed for further analyses. See **SI Appendix*, Materials and Methods* for detailed description of in vivo, ex vivo, tissue, and in vitro analyses.

### Collagen Endocytosis Assay.

For collagen endocytosis assay, sorted macrophages and fibroblasts were seeded in a 24-well plate and treated with 15 μg/mL of FITC-collagen for 24 h as in ref. 52. Cells were collected with stem pro accutase treatment (Gibco), washed, and stained for flow cytometry.

### Preparation of Proteomic Samples.

SAT samples were prepared as in ref. 77. Aliquots (30 µg) were trypsin digested using the modified filter-aided sample preparation method (78). Alternatively, 50 µg aliquots were filtered through 30 kDa molecular weight cut-off centrifuge filters to purify endogenous peptide fragments with masses <30 kDa. See **SI Appendix*, Materials and Methods* for detailed description of proteomic sample preparation.

## Supplementary Material

Appendix 01 (PDF)

Dataset S01 (XLSX)

Dataset S02 (XLSX)

Dataset S03 (XLSX)

## Data Availability

The mass spectrometry proteomics data have been deposited to the ProteomeXchange Consortium via the PRIDE partner repository with the dataset identifier PXD043721 (79). All other data are included in the manuscript and/or supporting information.
