# Supplementary material for: A macrophage-collagen fragment axis mediates subcutaneous adipose tissue remodeling in mice
Source: Proc Natl Acad Sci U S A. 2024 Feb 1;121(6):e2313185121. doi: 10.1073/pnas.2313185121 (PMC10861897; doi:10.1073/pnas.2313185121)
Supplement: Supplementary file 1 — Appendix 01 (PDF) [file pnas.2313185121.sapp.pdf]

PNAS

Supporting Information for

A Macrophage-Collagen Fragment Axis Mediates Subcutaneous Adipose Tissue Remodeling in Mice

Milica Vujičić, Isabella Broderick, Pegah Salmantabar, Charlène Perian, Jonas Nilsson, Carina Sihlbom Wallem, Ingrid Wernstedt Asterholm

Ingrid Wernstedt Asterholm
Email: IWA@neuro.gu.se

This PDF file includes:

Supporting Materials and Methods
Figures and legends S1 to S4
Tables S1 to S2

Other supporting materials for this manuscript include the following:

Datasets S1 to S3

Supporting Information Appendix

MATERIALS AND METHODS

Mouse studies

All experimental protocols for mouse studies were approved by Animal Ethics Committee at the Administrative Court of Appeals in Gothenburg, Sweden (1357/19 and 2733/20). Six-week-old male C57BL/6N wild type mice were purchased from Janvier Labs (Le Genest-Saint-Isle, France) and Charles River Laboratories (Sulzfeld, Germany). Vendor-matched mice were used in all experiments. All mice (up to 5 or 10 in small or big cages, respectively) were housed in the animal facility at Sahlgrenska Academy, University of Gothenburg, under standard laboratory conditions (12h light/dark cycle, lights on from 7 am), temperature (24°C) and *ad libitum* access to water and standard laboratory chow diet food (Special Diets Services). At the age of seven weeks, mice were randomly assigned to groups and fed either standard chow or high-fat diet (HFD, 60% Fat, 20% Protein and 20% Carbohydrate, D12492, Research Diets Inc., New Brunswick, NJ, USA) for one week, after which they were sacrificed for further analyses. For proliferation studies, mice were i.p. injected with 10 mg/kg EdU (5-ethynyl-2´-deoxyuridine, Invitrogen, Carlsbad, CA, USA) 24h prior sacrifice. For depletion of SAT macrophages, mice received 40 mg/kg of clodronate-loaded liposomes (Liposoma BV, Amsterdam, The Netherlands) directly into one subcutaneous fat pad and control PBS-loaded liposomes into the other fat pad on two occasions; day 0 and day 3 of the one-week HFD course. For CD206 neutralization studies, anti-mouse CD206 or IgG control (1 mg/kg) (R&D Systems, Minneapolis, MN, USA) were injected every other day into the space between subcutaneous fat-pad and skin (to avoid tissue injury), during aHFD challenge. The number of mice used for each experiment varied between 5-8 per group. The exact number of mice is indicated in the figure legends.

Cell culture and cell lines

Primary macrophages and fibroblasts were kept in DMEM supplemented with 10% FBS, 100 u/ml penicillin and 100 µg/ml streptomycin at 37°C in a 5% CO2 humid atmosphere. Mouse pre-adipocyte cell line 3T3-L1 (ZSenBio, Durham, NC, U.S.A.) was maintained in high-glucose DMEM (Thermo Fisher Scientific, MA, U.S.A) supplemented with 1% penicillin-streptomycin (Thermo Fisher) and 10% newborn calf serum (NBCS, Thermo Fisher). Differentiation into mature adipocytes was carried out according to standard procedures (1). In brief, after reaching confluency, cells were treated with differentiation cocktail containing 1 µm dexamethasone, 850 nM insulin, and 0.5 mM 3-isobutyl-1-methylxanthine suspended in DMEM 10% fetal bovine serum (FBS, Thermo Fisher) 1% penicillin-streptomycin. After two days, the media was exchanged for second differentiation cocktail containing 850 nM insulin. Two days after that, media was replaced to standard supplemented DMEM in which cells were kept for an additional 3 days. Maturity of 3T3-L1 adipocytes was determined visually, by confirming the presence of moderate to large lipid droplets.

L-929 conditioned media

The L-929 fibroblast cell line (ATCC, VA, U.S.A) was cultured in DMEM containing high glucose and supplemented with 10% FBS, 1% Non-essential amino acids (NEAA, Thermo Fisher Scientific), 1% penicillin–streptomycin RPMI. Cells were cultured in the same media for 4 days, and collected media was sterile-filtered (0.2 µm), aliquoted and stored at -80°C until use.

Isolation of bone-marrow stem cells, macrophage differentiation and polarization
Femur and tibia bones were dissected and rinsed in ethanol. The ends of the bones were aseptically cut, and the bone marrow cells were flushed out with RPMI using a syringe and needle (26G). To ensure single cell suspension, cells were gently pipetted and pelleted (500g, 5min). Red blood cells were removed by adding RBC lysis buffer (Biolegend, San Diego, CA, USA) for 5min at room temperature. Lysis was stopped by adding cell culture media and after washing step (500g, 5min) cells were resuspended in low glucose DMEM supplemented with 30% L-929 conditioned media, 1% non-essential amino acids (NEAA, Thermo Fisher). Cells were allowed to differentiate for 6 days. Differentiation into macrophages was confirmed by light microscopy, and cells were detached with accutase treatment and seeded in appropriate numbers into experimental dishes. Polarization to M2-like macrophages was conducted by supplementing the media with 10 ng/ml of mouse recombinant IL-4 (Thermo Fisher). Successful polarization was confirmed after 2 days with light microscopy and elongated cell morphology. For metabolic activation, M2 BMDM were stimulated with 25mM glucose, 0.5mM palmitate-BSA and 10nM insulin (2).

Sample collection
SAT, EWAT, liver and spleen were dissected, weighed and snap frozen in liquid nitrogen and kept at -80°C until further analyses; collected for histological analysis (see below) or collected for stroma vascular fraction (SVF) isolation (see below). Fasted trunk blood (serum) was collected.

Preparation of proteomic samples
SAT samples were homogenized on a Fast Prep-24 instrument (MP Biomedicals) for five repeated 40 s cycles at 6.5 m/s in 300 µl lysis buffer containing 2% sodium dodecyl sulfate (SDS), 50mM triethylammonium bicarbonate (TEAB). Lysed samples were cooled in +6°C for 1h and centrifuged in +4°C at 20 000 x g for 15 min and 200 µl of the supernatants, excluding the top fat layer, were transferred to new tubes. The cold centrifugation and transfer steps were performed two more times to get a clear supernatant of 125 µl. Protein concentrations were determined using Pierce BCA Protein Assay Kit (Thermo Fisher Scientific) and the Benchmark Plus microplate reader (Bio-Rad Laboratories) with bovine serum albumin (BSA) solutions as standards.

Aliquots (30 µg) were trypsin digested using the modified filter-aided sample preparation (FASP) method (3). Alternatively, 50 µg aliquots, were filtered through 30 kDa molecular weight cut-off centrifuge filters to purify endogenous peptide fragments with masses <30 kDa. For FASP, the samples were reduced at 56°C for 30 min in the lysis buffer with dithiothreitol (DTT) at 100 mM final concentration; and then diluted to 1:4 with 8 M urea, transferred onto Microcon-30kDa centrifugal filter units (Merck) and washed repeatedly with 8 M urea and once with digestion buffer (DB, 0.5% sodium deoxycholate (SDC) in 50 mM TEAB). Cysteine residues were modified using 10 mM methyl methanethiosulfonate (MMTS) in DB for 20 min at room temperature and the filters were washed twice with 100 µl DB. Pierce MS grade trypsin (Thermo Fisher Scientific) in DB was added at a protein ratio of 1:100 and reacted at 37°C overnight, and then an additional portion of trypsin was added and reacted for 4 h. The digested peptides were collected by centrifugation. For peptide fragment purification, the filtered samples were reduced at 60°C for 20 min in 5 mM tris(2-carboxyethyl)phosphine (TCEP) in the lysis buffer and then centrifuged at 10 000 g for 30 min using Microcon-30kDa centrifugal filter units (Merck), and cysteines derivatized with 10 mM MMTS for 20 min at room temperature.

Isobaric labelling was performed using Tandem Mass Tag (TMT-11plex) reagents (Thermo Fisher Scientific) according to the manufacturer's instructions. The labelled FASP samples were multiplexed into one pooled sample, and the same was done for the peptide fragment samples; concentrated using vacuum centrifugation, and SDC removed by acid precipitation with 10% TFA and subsequent centrifugation (FASP samples); or SDS removed with the HiPPR detergent removal spin column kit (Thermo Fisher Scientific) according to the manufacturer's instructions; and remaining SDC removed by acid precipitation with 10% TFA and centrifugation (peptide fragment samples). Samples were desalted with Pierce peptide desalting spin columns (Thermo Fisher Scientific) according to the manufacturer's protocol.

Pooled FASP samples were pre-fractionated into 40 primary fractions with basic pH reversed-phase chromatography (bRP-LC) using a Dionex Ultimate 3000 UPLC system (Thermo Fisher Scientific). Peptide separations were performed using a reversed-phase XBridge BEH C18 column (3.5 µm, 3.0x150 mm, Waters Corporation) and a linear gradient from 3% to 40% solvent B over 18 min followed by an increase to 100% B over 5 min and 100% B for 5 min at a flow of 400 µL/min. Solvent A was 10 mM ammonium formate buffer at pH 10 and solvent B was 90% acetonitrile, 10% 10 mM ammonium formate at pH 10. The fractions were concatenated into 10 fractions, dried and reconstituted in 3% acetonitrile, 0.2% formic acid.

LC-MS/MS analysis

FASP sample fractions and peptide fragment samples were analyzed on Orbitrap Lumos or Eclipse Tribrid mass spectrometers interfaced with the Easy-nLC1200 liquid chromatography system (Thermo Fisher Scientific). Peptides were trapped on an Acclaim Pepmap 100 C18 trap column (100 µm x 2 cm, particle size 5 µm, Thermo Fisher Scientific) and separated on an in-house packed analytical column (75 µm x 40 cm, particle size 3 µm, Reprosil-Pur C18, Dr. Maisch) using a gradient from 5% to 12% B over 5 min, 12% to 35% B over 72 min followed by an increase to 100% B for 3 min, and 100% B for 10 min at a flow of 300 nL/min. Solvent A was 0.2% formic acid and solvent B was 80% acetonitrile, 0.2% formic acid. MS scans were performed at 120 000 resolution, m/z range 375-1375. MS/MS (MS2) analysis was performed data-dependent with a cycle time of 3 s for the most intense 2+ to 7+ precursor ions. Precursor ions were isolated in the quadrupole with a 0.7 m/z isolation window with dynamic exclusion set to 10 ppm and 45 s duration. Isolated precursor ions were subjected to collision induced dissociation (CID) at 35% normalized collision energy (NCE) with a maximum injection time of 50 ms. The MS2 fragment ions were detected in the ion trap followed by multi-notch isolation of the 10 most abundant fragment ions for further fragmentation (MS3) by higher-energy collision dissociation (HCD) at 65% NCE and detection in the Orbitrap at 50 000 resolution in the m/z range 100-500. In addition, an HCD-MS2 methodology (MS2-TMT detection) for the peptide fragment sample was used on the Eclipse instrument with the alternative settings: m/z range 375-1500; NCE 38% with a maximum injection time of 150 ms and detection in the Orbitrap at 50 000 resolution; first mass m/z 100; and a dynamic exclusion duration of 12 s. The collagen fragment abundance data from both regular TMT (Dataset S2) and MS2-TMT methodologies (Dataset S3) were combined in Figure 4F and Dataset S1; the methodology giving the highest abundance were used when fragments were detected with both methodologies.

Proteomic data Analysis

The data files were merged for identification and relative quantification using Proteome Discoverer version 2.4 (Thermo Fisher Scientific). The mouse entries of the Swissprot database or selected collagen entries (35 sequences, February 2021) were used for the

database searches, using the Mascot search engine v. 2.5.1 (Matrix Science) with a MS tolerance of 5 ppm and fragment ion tolerance of 0.6 Da. Semitryptic peptides were accepted with zero missed cleavages, tryptic peptides were accepted with 3 missed cleavages and methionine oxidation, proline oxidation and Asn to Asp conversion were set as variable modification. For the peptide fragment samples, TMT on lysine (with and without oxidation) was set as variable modification. Cysteine methylation, and TMT on the peptide N-terminals (and on lysines for the FASP samples) were set as fixed modifications. Percolator was used for peptide spectral matches (PSM) validation with a false discovery rate (FDR) threshold of 1% and fixed values for the collagen database. Quantification was performed in Proteome Discoverer 2.4. The TMT reporter ions were identified with 3 mmu mass tolerance in the MS2 or MS3 HCD spectra and the TMT reporter S/N values for each sample were normalized within Proteome Discoverer 2.4 on the total peptide amount.

CCL-2 ELISA

To measure CCL-2 protein levels, animal serums were collected and analyzed with mouse CCL-2 ELISA kit (Abcam) per manufacturer's instructions.

RNA extraction and quantitative real-time PCR

Tissue RNA was isolated using commercial kit (ReliaPrepTM RNA Cell Miniprep System; Promega, Madison, WI, USA) and cDNA was prepared using High capacity RNA-to-DNA kit (Life Technologies, Carlsbad, CA, USA) according to the manufacturer's instructions. PCR reactions were detected using SYBR Green (Life Technologies) and performed on QuantStudio 7 flex system (Applied Biosystems, Foster City, CA, USA) using the comparative threshold cycle (Ct) method for relative quantification. Beta actin (*Actb*) and TATA-Box Binding Protein (*Tbp)* were used as endogenous controls. Primer sequences are summarized in Table S1. Data was analyzed in Quant Studio Real time PCR software v1.3.

Stroma vascular fraction and splenocyte Isolation

SAT, EWAT and spleens were collected and mechanically disrupted (spleen) by gentle teasing through 100 µm cell strainer (Thermo Fisher, Waltham, MA, USA) or minced to 2 mm^3 pieces (SAT and EWAT). Fat pads were digested in buffer containing 123 mM NaCl, 5 mM KCl, 5 mM CaCl2 1.2 mM KH2PO4, 1.2 mM MgSO4, 25 mM HEPES, 2 mM glucose, 200 nM adenosine, 1.5% BSA and 2 mg/ml collagenase type II (Sigma-Aldrich, St Louis, MO, USA) on 37°C with shaking (120 rpm). Collagenase activity was stopped with PBS 1 mM EGTA (ethylene glycol-bis(β-aminoethyl ether)-N,N,N′,N′-tetraacetic acid) on room temperature. Digest was filtered through 100 µm cell strainers and centrifuged for 5 min at 500g. Red blood cells from spleens, SAT and EWAT were removed with RBC lysis buffer (Biolegend, San Diego, CA, USA) treatment for 2 min at RT. Cells were washed, resuspended in DMEM 10% FBS and counted on automatic cell counter (Countess 2, Thermo Fisher), after which they were stained for flow cytometry analysis.

Peripheral blood mononuclear cell isolation

Mice or human blood was collected and diluted with PBS in 1:1 volume, in the presence of 2mM EDTA. Cells were pelleted (500g, 5min) and erythrocytes were removed with RBC lysis step. The resulting cell suspension was gently layered on density gradient (1.083 g/ml for mouse and 1.077 g/ml for human, Histopaque, Sigma Aldrich). Cells were centrifuged (400g, 20 min, no brakes), after which peripheral blood mononuclear cells

(PBMC) were collected, washed 2 times, and stained for flow cytometry (mice) or cultured for differentiation to macrophages.

Human PBMC-derived macrophages

PBMCs were cultured in RPMI 1640 (Sigma) supplemented with 10%, FBS 2mM L-Glutamine with addition of 50 ng/ml Macrophage Colony Stimulating Factor (M-CSF, Thermo Fisher). After 6 days, cells had visible macrophage morphology, with elongated shape and granulated cytoplasm. Macrophages were polarized to M2-like with IL-4 (20 ng/ml).

Flow cytometry

Cells were collected and counted on automatic cell counter. An equal number of cells were transferred to designated tubes and washed in PBS. Non-specific binding was blocked with anti-mouse CD16/CD32 antibody (Miltenyi Biotech, Bergisch Gladbach, Germany) in PBS 1% BSA for 15 min at +4°C. Cells were stained (45 min, at +4°C in dark) with fluorescently labelled antibodies. Dapi (Miltenyi) or LIVE/DEAD™ Fixable Blue Dead Cell Stain Kit (Thermo Fisher) was added as a dead cell marker for non-fixed and fixed cells, respectively. For EdU detection, Click-iT™ Plus EdU Alexa Fluor™ 488 Flow Cytometry Assay Kit (Thermo Fisher) was used per manufacturer's instructions. Events (20000) were acquired on FACSCANTO 2 flow cytometer (BD Bioscience, Franklin Lakes, NJ, USA). All fluorophores were compensated with compensation controls, and gates were set with Fluorescence Minus One (FMO) controls. Gating strategy: Cells were gated based on their FSC-SSC appearance, followed by selection of single live $CD45^+$ (leukocytes) or $CD45^-$ (fibroblasts) cells. The flow cytometry data was analyzed using FlowJo software version 10.6.0 or newer (FlowJo, LLC, Ashland, OR) and FACS Diva software version 7.0 (BD Bioscience). All antibodies used for flow cytometry are summarized in Table S2.

Histological analyses

Tissues were fixed in 4% buffered formalin for 48h at RT (VWR Chemicals, Stockholm, Sweden), followed with paraffin-embedding and cross-sectioning (7 µm). For fibrillar collagen measurements, Picrosirius Red Stain Kit (Polysciences, Warrington, PA, USA) was used per manufacturer's protocol. Briefly, after deparaffinization and rehydration, tissue sections were incubated with phosphomolybdic acid for 2 min, stained with picrosirius red solution for 90 min at RT, followed by 1 min rinse in HCl (2 times), dehydrated, cleared, and mounted. Images were obtained on Olympus BX60 light microscope (Olympus, Tokyo, Japan) equipped with Plan-Apo 20x/0.8 objective. For immunofluorescence labelling, following deparaffinization and rehydration, tissue sections were blocked with PBS 10% normal donkey serum (Abcam) for 1h at RT, and stained with primary antibodies (Table S2, 1:150 in incubation buffer: PBS 1% BSA 0.3% Triton-X) at +4°C overnight. After PBS washing, slides were incubated with donkey anti-rabbit Alexa 555 (1:500) secondary antibody (Thermo Fisher) for 40 min at RT in dark. After final wash, slides were mounted with vectashield antifade mounting medium with Dapi (Vector laboratories, Burlingame, CA, USA). Sections were visualized on LSM 700 laser scanning confocal microscope (Zeiss, Oberkochen, Germany) equipped with Plan-Apochromat 20x/0.8 objective. Dapi was excited with 405nm laser and Alexa Fluor 555 with 555nm laser. Image acquisition was performed with Zeiss Zen Black 2012 software. To identify non-specific staining of the secondary reagents, negative controls were included in all immunofluorescent staining experiments. Images were analyzed in Fiji v1.53c software.

Protein extraction and quantification

Total proteins in tissue were extracted by homogenization in RIPA buffer containing 150 mM sodium chloride, 1.0 % Triton X-100, 0.5 % sodium deoxycholate, 0.1 % sodium dodecyl sulfate, 50 mM Tris, pH 8.0 and supplemented with complete protease inhibitor cocktail (Roche, Basel, Switzerland). Protein concentration was determined with Pierce™ BCA Protein Assay Kit (Thermo Fisher) per manufacturer's instructions. Briefly, samples diluted 50x with PBS were loaded into 96-well plate in duplicates. Standard curve was prepared from serial dilutions of BSA stock (2mg/ml). Working solution was prepared as a mix of Solution A and Solution B in a 50:1 ratio and added on the samples. Samples were incubated at 37°C, 120 RPM for 30 minutes. Absorbance was measured on Spectramax i3x multiplate reader (Molecular Devices) on 562nm wavelength.

Western blot

Proteins (25-50µg) were mixed with 4x Laemmli Sample Buffer (Bio-Rad Laboratories, Hercules, CA, USA) with addition of reducing agent β-mercaptoethanol when necessary, and denaturized at 95°C for 5 min. After, samples were loaded onto Criterion-TGX stain free gels (Bio-Rad), ran on SDS-PAGE and transferred onto 0.2 µm PVDF transfer membranes (Bio-Rad). Membranes were stained for total protein load with MemCode reversible protein stain kit (Thermo Fisher) per manufacturer's instructions. Unspecific binding was blocked with PBS 5% BSA (1h at RT), and membranes were probed with primary antibodies overnight at +4°C. After wash in TBST, membranes were incubated with secondary HRP-conjugated antibodies for 1h at RT. Development of immunoblots was performed using an enhanced chemiluminescence kit (Bio-Rad). Bands were visualized on the ChemiDoc XRS system (Bio-Rad, CA, USA) and analyzed with the image analysis program Image Lab© (Bio-Rad, CA, USA). Antibodies used are summarized in Supplementary Table 5.

Magnetic-Activated Cell Sorting

To sort adipose tissue macrophages and fibroblasts, magnetic-activated cell sorting was applied. Briefly, isolated SVF cells were incubated (15 min on ice) with magnetic beads labelled with anti-mouse F4/80 (for macrophages), or anti-mouse CD45 and anti-mouse CD90.2 for fibroblasts (all Miltenyi Biotec). After washing, solutions were loaded onto ms-columns that were introduced to strong magnetic field. Macrophages were selected as the F4/80^{+} positive fraction and fibroblasts as F4/80^{-}CD45^{-}CD90.2^{+} fraction. The purity of isolates was confirmed by qPCR and flow cytometry.

Collagen type 1 labelling

Labelling of collagen type 1 was performed by modification of the previously described procedure (4). Acid extracted rat tail tendon collagen type I (3 mg/ml, Thermo Fisher) was neutralized and incubated overnight on 37°C to form a gel. Collagen gel was covalently labelled with 2 mg/ml FITC dye for 45 min at RT. To remove unbounded dye, gel was extensively washed with PBS for 3 days, followed by 2-day wash with dH_2O to remove salt crystals. Collagen was then denaturized in 20 mM HCl and stirred with magnetic stirrer at +4°C until turning into solution. Non-denaturized parts were removed by centrifugation (12000 g, 15 min, +4°C). Finally, FITC-labelled collagen was mixed with unlabeled collagen (1:5, final concentration 1.88 mg/ml) and this mix was used in all experiments.

Preparation of collagen gels

Collagen gels were prepared as in (5). Briefly, FITC-labelled collagen was neutralized with 10xPBS 100mM HEPES and DMEM 10% FBS with cells ($0.1x10^6$) inside, in an 8:1:1 ratio (final collagen concentration 2 mg/ml). Solution was casted in a 35mm dish with 20mm glass bottom well (Cellvis) and left to polymerize for 45 min at 37°C with 5% CO_2. Once

the gels formed, 2 ml of DMEM 10% FBS was added in the dish and left for 24h in the incubator. Lysosomes were stained with lysotracker red (90 min in lysotracker A solution followed by 30 min incubation in lysotracker B solution). Nuclei were stained with Dapi. Images were acquired with LSM 700 laser scanning confocal microscope (Zeiss, Oberkochen, Germany) equipped with Plan-Apochromat 20x/0.8 objective. Dapi was excited with 405nm laser, lysotracker red with 573nm laser and FITC-collagen with 493nm laser. Image acquisition was performed with Zeiss Zen Black 2012 software.

Antibody mediated CD206 neutralization *ex vivo*
Wild-type mouse SAT macrophages were sorted with magnetic beads ($F4/80^+$) and left overnight in DMEM 10% FBS. In the morning, media was changed to DMEM 0.5%BSA and cells were treated with FITC-collagen 15 µg/ml in the presence of anti-CD206 antibody or IgG control (10 µg/ml). After 3h cells were collected and stained for flow cytometry.

Cell viability
Cell viability was measured with Crystal violet method. Cells were allowed to attach into a 96-well plate. Wells were washed two times with PBS and incubated with 0.1% Crystal Violet solution (Sigma-Aldrich) for 15 min at room temperature. After thorough washing of unbound dye with dH2O, bounded dye was dissolved with 33% acetic acid. Absorbance was read on a SpectraMax i3x plate reader at 540 nm and 670 nm.

Oral glucose tolerance test (OGTT)
For basal glucose measurements, mice were fasted during the daytime for 4 hours, and blood samples were taken from the tail vein. After that, mice received an oral load of D-glucose (2.5 g/kg, Sigma Aldrich) dissolved in water. Blood glucose levels at 15, 30, 60 and 120min were measured using a glucometer (Contour from Bayer; Baser, Switzerland). Serum insulin levels were measured by a commercial ELISA kit (Mouse Insulin ELISA, Mercodia, Uppsala, Sweden) per manufacturer's instructions.

Hepatic Triglycerides measurement
Pieces of liver were weighed, and lipids were extracted by Folch method (6). Briefly, liver tissue was lysed in 2:1 chloroform:methanol, washed with 0.9M NaCl solution followed by collection of the lower phase. After overnight evaporation dry pellet was resuspended in isopropanol (Sigma) and triglycerides were measured with Triglyceride kit (Randox Laboratories Ltd, Crumlin, Northern Ireland, UK) per manufacturer's instructions.

Statistical analysis
All data are presented as mean±SEM. Statistical analyses were performed in GraphPad Prizm (9.4.1 and newer). Comparisons between the two groups were made with unpaired Student's t test. Parametric test was used for normally distributed data, otherwise Mann-Whitney test. For comparison between more than two groups, one-way and two-way ANOVA were used where appropriate, followed by Tukey and Bonferroni post-hoc analyses, respectively. For the proteomics data analysis, abundances were normalized, and log transformed. Comparisons were made with multiple t-tests which were corrected for multiple testing using the Benjamini-Hochberg correction, with significance being set at $p < 0.01$ at an FDR of 1%. Differentially abundant proteins were subjected to enrichment analyses in Cytoscape (version 3.8.2) using GO and KEEG ontologies. PCA analysis was conducted in R Studio (version 4.2.1 and newer). Data was tested for outliers with Grubb's outlier test ($p<0.05$).

Figure S1

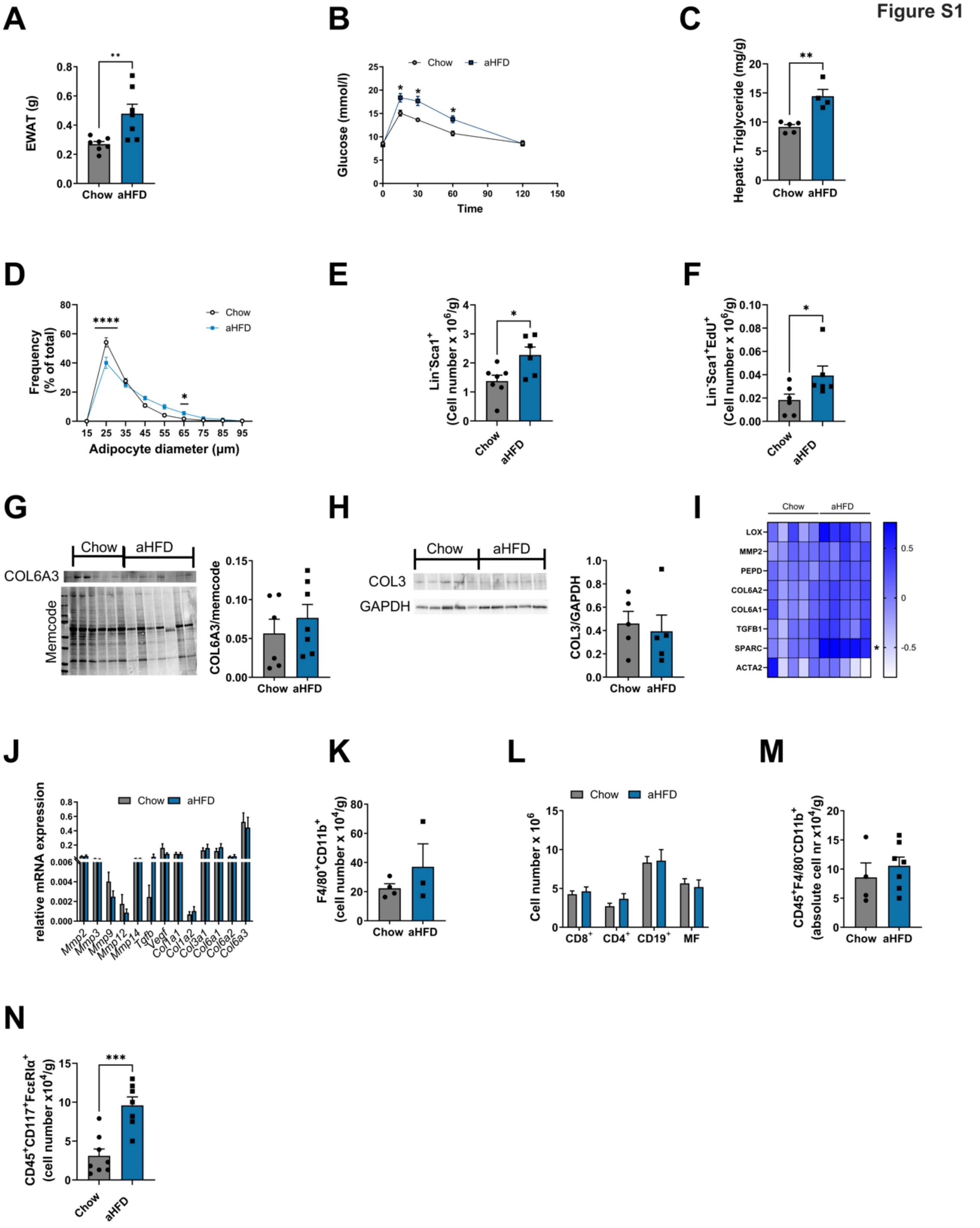

Fig. S1. Effect of aHFD on metabolism, ECM organization and immune cells. 7-weeks old mice were placed on chow or HFD for one week**.** (A) EWAT weight (n=7/group). (B) Oral glucose tolerance test (n=5/group). (C) Hepatic triglycerides (n=4-5 per group). (D) Adipocyte size distribution (n=4-5/group). Number of total (E) and proliferating (F) mesenchymal stem cells ($CD45^{-}CD31^{-}Sca1^{+}$, n=6-7/group) in SAT. Western blot analysis of (G) COL6A3 and (H) COL3 (n=5-7 per group). Proteins were normalized to (G) total protein content or (H) GAPDH. Absolute cell number per gram of tissue of (I) EWAT

macrophages (single live CD45$^+$F4/80$^+$CD11b$^+$) (n=3-4 per group). (J) Spleen cytotoxic T lymphocytes (CD8$^+$), T helper lymphocytes (CD4$^+$), B-cells (CD19$^+$) and macrophages (F4/80$^+$CD11b$^+$) (n=8/group), (K) SAT neutrophils (CD45$^+$F4/80$^-$CD11b$^+$, n=4-7/group), and (L) SAT mast cells (CD45$^+$CD117$^+$FcεRIα$^+$, n=7-8/group). Data is presented as mean±SEM. Unpaired student’s t tests (*p<0.05, **p<0.01, ***p<0.005, ****p<0.0001). **Related to Fig. 1.**

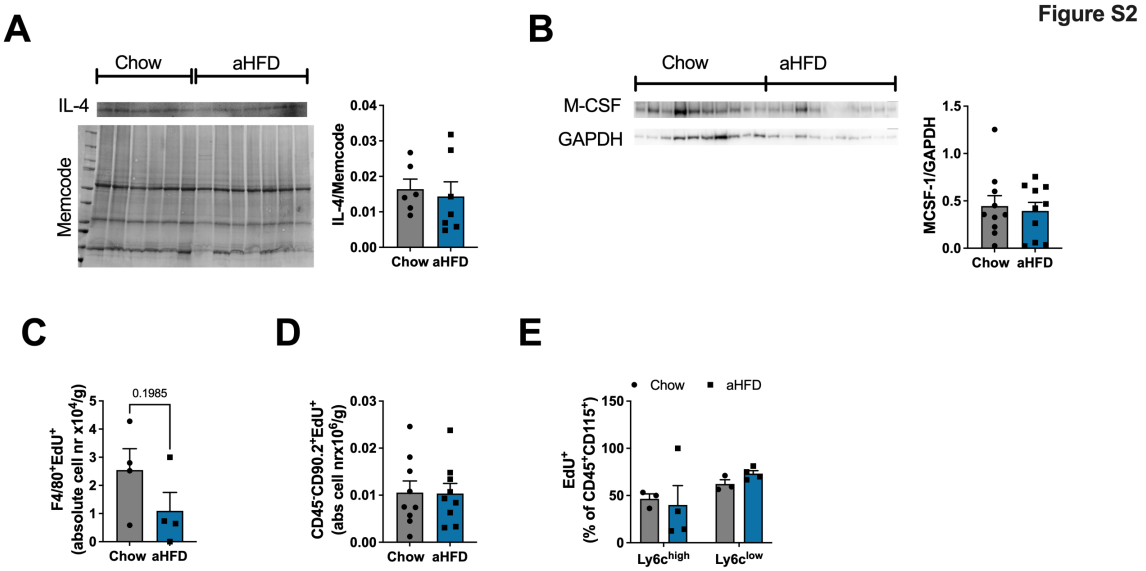

Fig. S2. Effect of aHFD on the proliferation of different cell types. (A) Western blot of IL-4 in SAT of chow (n=6) and aHFD (n=7) mice normalized to total protein content. (B) Western blot of MCSF-1 in SAT of chow (n=9) and aHFD (n=10) mice normalized to GAPDH levels. (C) Number of proliferating macrophages (F4/80$^+$EdU$^+$) per gram of EWAT of chow and aHFD mice (n=4/group). (D) Number of proliferating fibroblasts (CD45$^-$CD90.2$^+$EdU$^+$) per gram of SAT of chow and aHFD mice (n=9/group). (E) Percentage of proliferating (EdU$^+$) classical (Ly6c^{high}) and non-classical (Ly6c^{low}) monocytes (CD45$^+$CD115$^+$) from blood of chow and aHFD mice (n=3-4/group). Data is presented as mean±SEM. Unpaired student’s t tests. **Related to Fig. 2.**

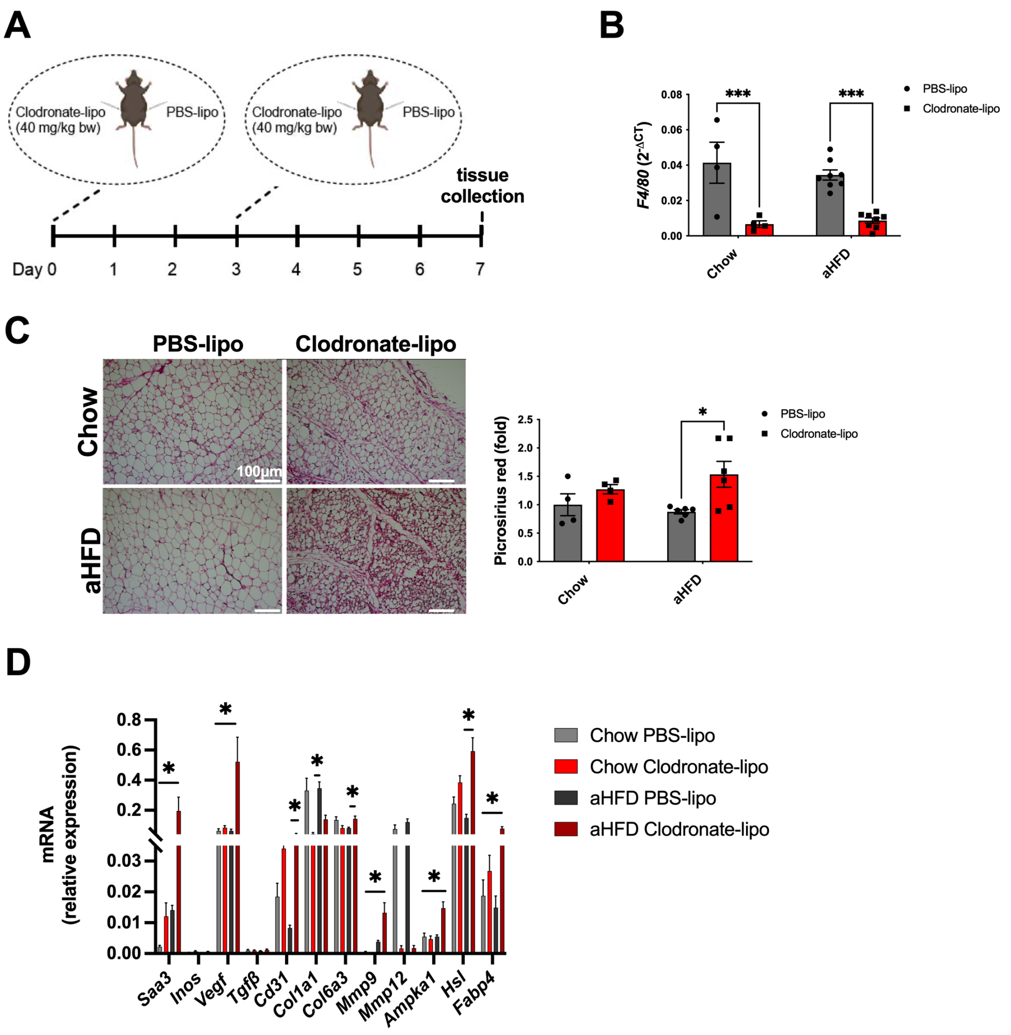

Fig. S3. SAT changes induced by clodronate depletion of macrophages. (A) Schematic presentation of clodronate experiment. (B) Expression of *F4/80* (relative to *Bactin*) in SAT of clodronate or PBS-treated chow or aHFD mice (n=4 or 8 per group). (C) Representative images (scale bar=100μm) and quantification of picrosirius red staining in SAT of clodronate or PBS-treated chow or aHFD mice (n=4-6 per group). Data are presented as fold change of chow PBS-treated mice. (D) Gene expression (relative to *Bactin*) in SAT of clodronate or PBS-treated chow or aHFD mice (n=4-6 per group). Data is presented as mean±SEM. 2-way ANOVA (*p<0.05, ***p<0.001, ****p<0.0001). **Related to Fig. 3.**

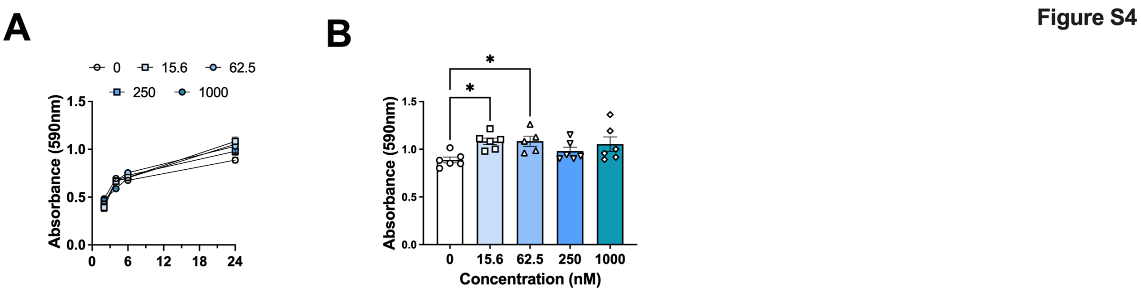

Fig. S4. [PPG]$_5$ effect on 3T3-L1 cells *in vitro*. (A-B) Viability of 3T3-L1 pre-adipocytes after treatment with different concentrations of collagen mimetic peptide (n=6 per group). Data is presented as mean±SEM. 1-way ANOVA (*p<0.05). **Related to Fig. 5.**

Table S1. List of primer sequences.

Gene	Forward 5'-3'	Reverse 5'-3'
Bactin	GACCCAGATCATGTTTGAGA	GAGCATAGCCCTCGTAGAT
F4/80	CTTTGGCTATGGGCTTCCAGTC	GCAAGGAGGACAGAGTTTATCGTG
Saa3	TAAAGTCATCAGCGATTCCAGAG	CAACCCAGTAGTTGCTCCTCTTC
Inos	TGCTGTTCTCAGCCCAACAA	GAACTCAATGGCATGAGGCA
Vegf	GTCCGATTGAGACCCTGGTG	GCTGGCTTTGGTGAGGTTTG
Tgfb	GTGAATGGCTCTCCTTCGAC	CCTCGAGCTCTTCGCTTTTA
Cd31	ACTTCTGAACTCCAACAGCGA	CCATGTTCTGGGGGTCTTTAT
Col1a1	GCTCCTCTTAGGGGCCACT	CCACGTCTCACCATTGGGG
Col1a2	GTAACTTCGTGCCTAGCAACA	CCTTTGTCAGAATACTGAGCAGC
Col3a1	CCTGGCTCAAATGGCTCAC	CAGGACTGCCGTTATTCCCG
Col6a1	TGCCCTGTGGATCTATTCTTCG	CTGTCTCTCAGGTTGTCAATG
Col6a2	CTACTCACCCCAGGAGCAGGAA	TCAACGTTGACTGGGCGATCGG
Col6a3	AACCCTCCACATACTGCTAATTC	TCGTTGTCACTGDCTTCATT
Mmp2	ACCTGAACACTTTCTATGGCTG	CTTCCGCATGGTCTCGATG
Mmp3	ACATGGAGACTTTGTCCCTTTTG	TTGGCTGAGTGGTAGAGTCCC
Mmp9	CTGGACAGCCAGACACTAAAG	CTCGCGGCAAGTCTTCAGAG
Mmp12	CCCTCGATGTGGAGTGCCCG	TCCTCACGCTTCATGTCCGGAGT
Mmp14	CCCGCCTATGGGCCCAACAT	CCCGCCAGAACCATCGCTCC
Ampka1	AAGCCGACCCAATGACATCA	CTTCCTTCGTACACGCAAAT
Hsl	TTCGAGGGTGATGAAGGACT	ACTCTGGGTCTATGGCGAAT
Fabp4	GAACCTGGAAGCTTGTCTTCG	ACCAGCTTGTCACCATCTCG
Ccl2	ACTGAAGCCAGCTCTCTCTTCC	TTCCTTCTTGGGGTCAGCACAG
Il1b	AATGAAAGACGGCACACCCA	TGCTTGTGAGGTGCTGATGT
Il6	GACAAAGCCAGAGTCCTTCAGA	GTCTTGGTCCTTAGCCACTCC
Lox	TCTTCTGCTGCGTGACAACC	GAGAAACCAGCTTGGAACCAG
Pcna	CCCAGAACAGGAGTACAGCTG	TGTTCCCATTGCCAAGCTCT
Cd206	TGTGGTGAGCTGAAAGGTGA	CAGGTGTGGGCTCAGGTAGT
Tnfa	CAGGCGGTGCCTATGTCTC	CGATCACCCCGAAGTTCAGTAG
Cd11c	CTGGATAGCCTTTCTTCTGCTG	GCACACTGTGTCCGAACTC
Il10	GCTCTTACTGACTGGCATGAG	CGCAGCTCTAGGAGCATGTG

Table S2. List of antibodies and reagents.

ANTIBODIES	SOURCE	IDENTIFIER
APC/Cyanine7 anti-mouse CD45	BioLegend	Cat#103115
F4/80 Monoclonal Antibody (BM8), PE-Cyanine7	Thermo Fisher Scientific	Cat#25-4801-82
PerCP/Cyanine5.5 anti-mouse/human CD11b	Biolegend	Cat# 101228
CD11c Monoclonal Antibody (N418), FITC, eBioscience	Thermo Fisher Scientific	Cat# 11-0114-82
CD11c-FITC	Miltenyi Biotec	Cat# 130-102-466
APC anti-mouse CD206 (MMR)	BioLegend	Cat# 141708
Alexa Fluor(R) 488 anti-mouse CD206 (MMR)	BioLegend	Cat# 141710
CD192 (CCR2) Antibody, anti-mouse, PE, REAfinity™	Miltenyi Biotec	Cat# 130-117-548
Alexa Fluor(R) 488 anti-mouse CX3CR1	BioLegend	Cat# 149021
CD192 (CCR2) Antibody, anti-mouse, APC, REAfinity™	Miltenyi Biotec	Cat# 130-119-658
CX3CR1 Monoclonal Antibody (2A9-1), PE, eBioscience	Thermo Fisher Scientific	Cat# 12-6099-42
CD90.2 Antibody, anti-mouse, PE	Miltenyi Biotec	Cat# 130-102-489
CD140a Antibody, anti-mouse, APC	Miltenyi Biotec	Cat# 130-102-473
CD115 (c-fms) Monoclonal Antibody (AFS98), APC, eBioscience	Thermo Fisher Scientific	Cat# 17-1152-82
Ly-6C Monoclonal Antibody (HK1.4), PerCP-Cyanine5.5, eBioscience	Thermo Fisher Scientific	Cat# 45-5932-82
PCNA Antibody, anti-human/mouse/rat, APC, REAfinity™	Miltenyi Biotec	Cat# 130-114-704
CD8a Monoclonal Antibody (53-6.7), APC, eBioscience	Thermo Fisher Scientific	Cat# 17-0081-82
CD4 Monoclonal Antibody (GK1.5), FITC, eBioscience	Thermo Fisher Scientific	Cat# 11-0041-82
CD19 Monoclonal Antibody (eBio1D3 (1D3)), PerCP-Cyanine5.5, eBioscience	Thermo Fisher Scientific	Cat# 45-0193-82
CD3 Monoclonal Antibody (REA613), APC-Vio 770	Miltenyi Biotec	Cat# 130-113-698
CD19 Antibody, anti-human (REA675), APC-Vio® 770, REAfinity™	Miltenyi Biotec	Cat# 130-114-169
CD56 Antibody, anti-human (REA196), APC-Vio® 770, REAfinity™	Miltenyi Biotec	Cat# 130-114-739
CD66b Antibody, anti-human (REA306), APC-Vio® 770, REAfinity™	Miltenyi Biotec	Cat# 130-120-146
APC/Cyanine7 anti-human CD1c (L161)	BioLegend	Cat# 331520
CD11b Antibody, anti-human (REA713) PerCP-Vio® 700, REAfinity™	Miltenyi Biotec	Cat# 130-110-615
CD206 Antibody, anti-human (DCN228) APC	Miltenyi Biotec	Cat# 130-124-012
CD117 Antibody, anti-mouse (3C11) APC	Miltenyi Biotec	Cat# 130-122-948
FcεRIα Antibody, anti-mouse (MAR1) PE	Miltenyi Biotec	Cat# 130-102-351
CD31 APC/Fire(TM) 750 anti-mouse (MEC13.3)	BioLegend	Cat# 102528
Ly-6A/E (Sca-1) Monoclonal Antibody (D7), PE-Cyanine7	Thermo Fisher Scientific	Cat# 25-5981-82

Anti-Collagen type I, cleavage site (Col1-¾C), ImmunoGlobe	ImmunoGlobe	Cat# 0217-050
Anti-Collagen III Polyclonal Antibody	Thermo Fisher Scientific	Cat# PA5-27828
Anti-COL6a3 antibody	MyBioSource	Cat# MBS2027727
Anti-Collagen I Antibody	Novus	Cat# NB600-408
Picrosirius Red Stain Kit	Polysciences	Cat# 24901-500
Mouse MMR/CD206 Affinity Purified Polyclonal Ab	R and D Systems	Cat# AF2535
Goat Anti-Goat Normal IgG Control antibody, Unconjugated	R and D Systems	Cat# AB-108-C
Rat Anti-Mouse M-csf Monoclonal antibody, Unconjugated, Clone 131614	R and D Systems	Cat# MAB4161
Anti-CD31 MicroBeads	Miltenyi Biotec	Cat# 130-097-418
Anti-CD45 MicroBeads, mouse	Miltenyi Biotec	Cat# 130-052-301
Anti-F4/80 MicroBeads UltraPure, mouse	Miltenyi Biotec	Cat# 130-110-443
Anti-CD90.2 MicroBeads, mouse	Miltenyi Biotec	Cat# 130-121-278
Anti-beta Actin antibody [8F10-G10]	Abcam	Cat# ab170325
Donkey anti-Rabbit IgG (H+L) Highly Cross-Adsorbed Secondary Antibody, Alexa Fluor™ 555	Thermo Fisher Scientific	Cat# A-31572
Donkey polyclonal Secondary Antibody to Rabbit IgG - H&L (HRP)	Abcam	Cat# ab6802
Donkey polyclonal Secondary Antibody to Goat IgG - H&L (HRP)	Abcam	Cat# ab6885
Rabbit anti-Mouse IgG H&L (HRP) secondary antibody	Abcam	Cat# ab97046
Rat Anti-Mouse Il-4 Monoclonal antibody, Unconjugated, Clone 30340	R and D Systems	Cat# MAB404
IkappaBalpha Antibody	Cell Signaling Technology	Cat#9242
**CHEMICALS, PEPTIDES, RECOMBINANT PROTEINS**		
Recombinant Mouse IL-4 (carrier-free)	BioLegend	Cat# 574302
Mouse IFN-gamma Recombinant Protein	Thermo Fisher Scientific	Cat# PMC4031
Human M-CSF Recombinant Protein	Thermo Fisher Scientific	Cat # PHC9501
Recombinant Human IL-4 Protein, CF	R and D Systems	Cat# BT-004-010
GlutaMAX™ Supplement	Thermo Fisher Scientific	Cat# 35050061
Generic collagen fragment	Thermo Fisher Scientific	Cat# A8271-2
Collagenase, Purified CLSPA lot nr 50N20658	Worthington	Cat# LS005273
Recombinant Human MMP-13 Protein, CF	R and D Systems	Cat# 511-MM-010
Mca-PLGL-Dpa-AR-NH2 Fluorogenic MMP Substrate	R and D Systems	Cat# ES001
Collagen I Rat Protein, Tail	Thermo Fisher Scientific	Cat# A1048301
Collagenase from Clostridium histolyticum	Sigma Aldrich	Cat# C6885-1G
FcR Blocking Reagent, mouse	Miltenyi Biotec	Cat# 130-092-575

DAPI Staining Solution	Miltenyi Biotec	Cat# 130-111-570
MEM Non-Essential Amino Acids Solution	Thermo Fisher Scientific	Cat# 11140050
EdU (5-ethynyl-2'-deoxyuridine)	Thermo Fisher Scientific	Cat# A10044
Clodronate liposomes and control liposomes (PBS)	Liposoma	Cat# CP-005-005
Lipopolysaccharide (LPS)	Thermo Fisher Scientific	Cat# 00-4976-93
EDTA (Ethylenediaminetetraacetic acid tetrasodium salt dihydrate)	Amresco	Cat# 0322
EGTA (Ethylene glycol-bis(2-aminoethylether)-N,N,N′,N-tetraacetic acid)	Sigma Aldrich	Cat# E4378
Normal Donkey Serum	Abcam	Cat# ab7475
RBC Lysis Buffer	BioLegend	Cat# 420301
Rabbit serum	Sigma Aldrich	Cat# R9133
Normal Goat Serum	Thermo Fisher Scientific	Cat# 31872
Clarity Western ECL Substrate	Bio Rad	Cat# 1705061
RIPA buffer	Abcam	Cat# ab156034
cOmplete tablets	Roche	Cat# 4693159001
PhosSTOP	Roche	Cat# 4906845001
BSA	Sigma Aldrich	Cat# A7906
Free fatty acid BSA	Sigma Aldrich	Cat# A7030
Fluorescein isothiocyanate isomer I	Sigma Aldrich	Cat# F7250
**COMMERCIAL ASSAYS**		
Mouse CCL2/JE/MCP-1 Quantikine ELISA Kit	R and D Systems	Cat# MJE00B
Cell Navigator® Lysosome Staining Kit *Deep Red Fluorescence*	AAT Bioquest	Cat# 22659
LIVE/DEAD™ Fixable Blue Dead Cell Stain Kit	Thermo Fisher Scientific	Cat# L23105
Click-iT™ Plus EdU Alexa Fluor™ 488	Thermo Fisher Scientific	Cat# C10633
Pierce™ Reversible Protein Stain Kit for PVDF Membranes	Thermo Fisher Scientific	Cat# 24585
Pierce™ BCA Protein Assay Kit	Thermo Fisher Scientific	Cat# 23225
ReliaPrep™ RNA Miniprep Systems	Promega	Cat# Z6112
Fast SYBR™ Green Master Mix	Thermo Fisher Scientific	Cat# 4385618
High-Capacity cDNA Reverse Transcription Kit	Thermo Fisher Scientific	Cat# 4368814
Randox Triglycerides (GPO-PAP)	Randox Laboratories Ltd	Cat# TR210
Mouse Insulin ELISA	Mercodia	Cat# 10-1247-01

Datasets S1 to S3 (separate excel file)
Dataset S1. Collagen fragments in SAT. Related to Fig. 4.
Dataset S2. Collagen fragments in SAT as judged by TMT detection only. Related to Fig.4.
Dataset S3. Collagen fragments in SAT as judged by MS2 TMT detection only. Related to Fig. 4.

References

1. E. Peris *et al.*, Antioxidant treatment induces reductive stress associated with mitochondrial dysfunction in adipocytes.
2. M. Kratz *et al.*, Metabolic dysfunction drives a mechanistically distinct proinflammatory phenotype in adipose tissue macrophages. *Cell Metab* **20**, 614-625 (2014).
3. J. R. Wisniewski, A. Zougman, N. Nagaraj, M. Mann, Universal sample preparation method for proteome analysis. *Nat Methods* **6**, 359-362 (2009).
4. H. Birkedal-Hansen *et al.*, Matrix metalloproteinases.
5. B. Burkel *et al.*, Preparation of 3D Collagen Gels and Microchannels for the Study of 3D Interactions In Vivo. *J Vis Exp* 10.3791/53989 (2016).
6. J. Folch, M. Lees, G. H. S. Stanley, A Simple Method for the Isolation and Purification of Total Lipides from Animal Tissues. *Journal of Biological Chemistry* **226**, 497-509 (1957).
